# Supplementary material for: Different adjuvanted pediatric HIV envelope vaccines induced distinct plasma antibody responses despite similar B cell receptor repertoires in infant rhesus macaques
Source: PLoS One. 2021 Dec 31;16(12):e0256885. doi: 10.1371/journal.pone.0256885 (PMC8719683; doi:10.1371/journal.pone.0256885)
Supplement: S1 Fig — A-J. Representative flow cytometry analyses for single cell HIV Env-specific memory B cell sorting from PBMC and tissues of infants by vaccination groups. Percentage of memory B cells (CD20+CD27+IgD-), which were reactive to HIV 1086c gp120 K160N tagged with two colors-BV421 and AF647 (double positive cells) indicated in the upper right quadrant. Gates were drawn based on isotype controls and fluorescent minus one (FMO) controls. (PDF) [file pone.0256885.s001.pdf]

S1 Fig

A. HIV Env+adjuvant group, animal 45519, PBMC (W10), gating strategy: lymphocytes → Singlets (SSC or FSC) → Live-dead → CD14-CD16- → CD3-CD20+ → IgD-CD27all → 1086c.gp120 double positive

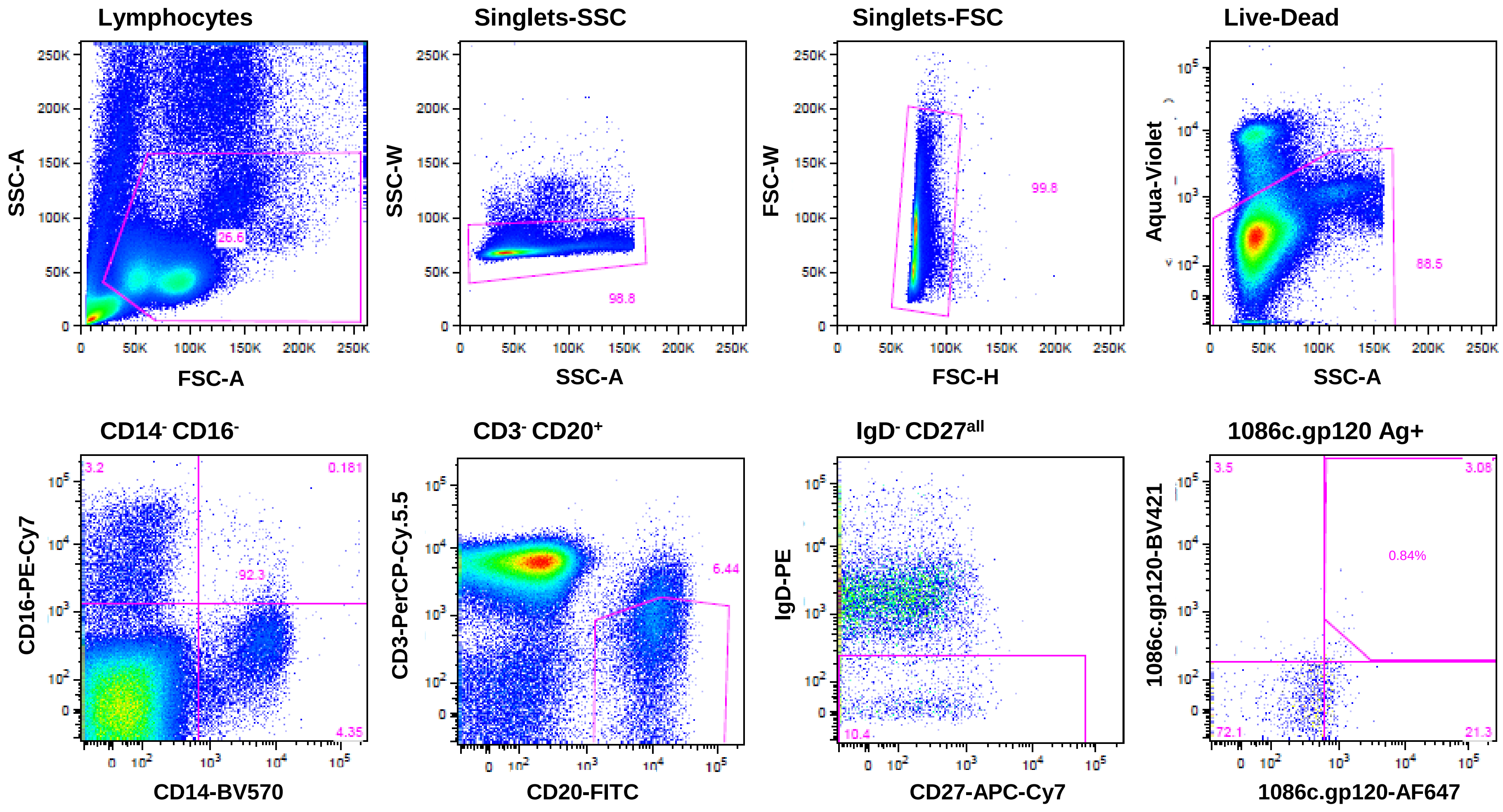

S1 Fig

B. HIV Env+adjuvant group, animal 45521, Spleen (necropsy), representative data of 2 independent sort, gating strategy: lymphocytes → Singlets (SSC or FSC) → Live-dead → CD14-CD16- → CD3-CD20+ → IgD-CD27all → 1086c.gp120 double positive

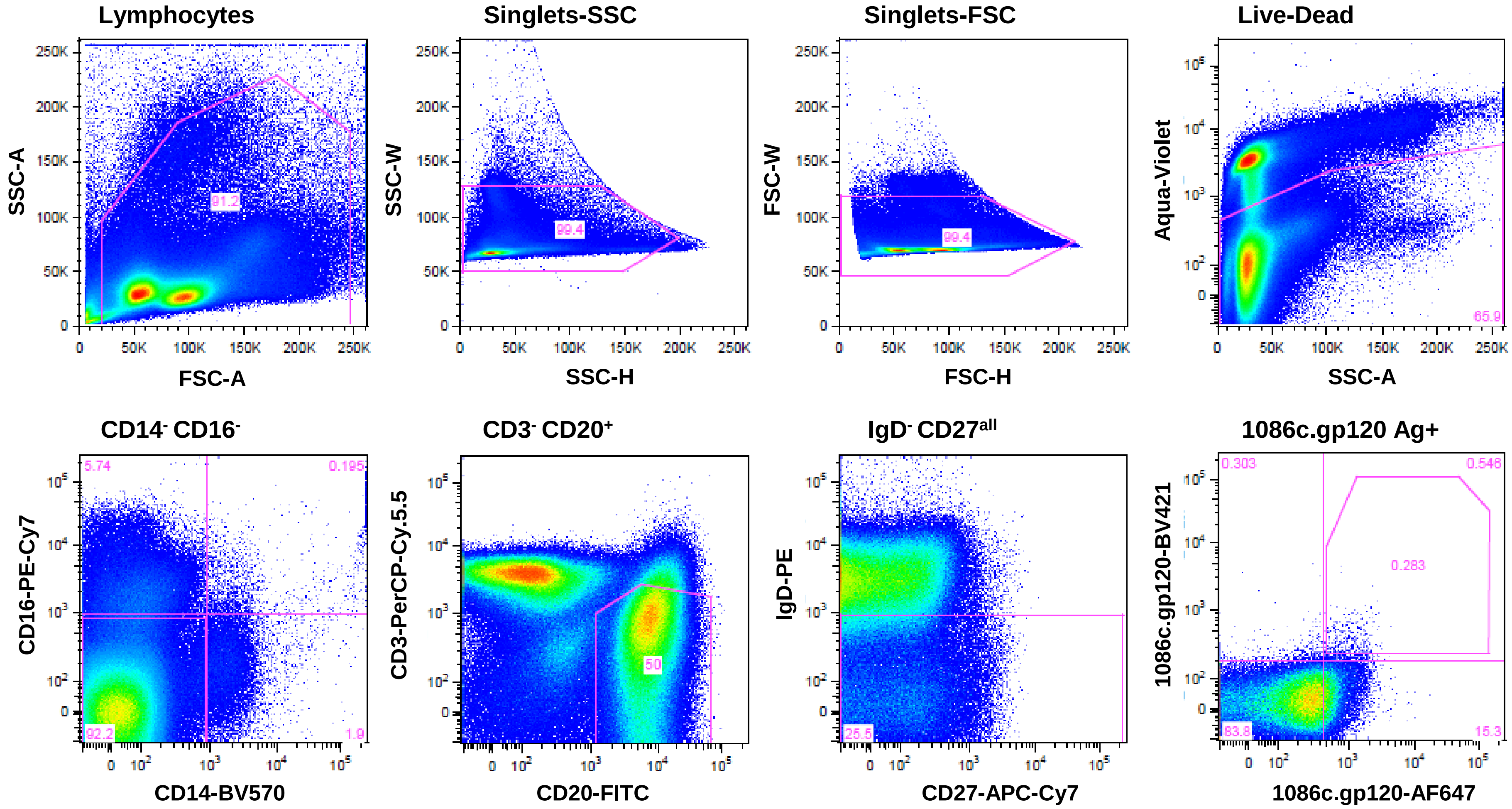

S1 Fig

C. HIV Env+adjuvant group, animal 45522, Oral LN (retropharengeal, necropsy), representative data of 3 independent sort, gating strategy: lymphocytes → Singlets (SSC or FSC) → Live-dead → CD14-CD16- → CD3-CD20+ → IgD-CD27all → 1086c.gp120 double positive

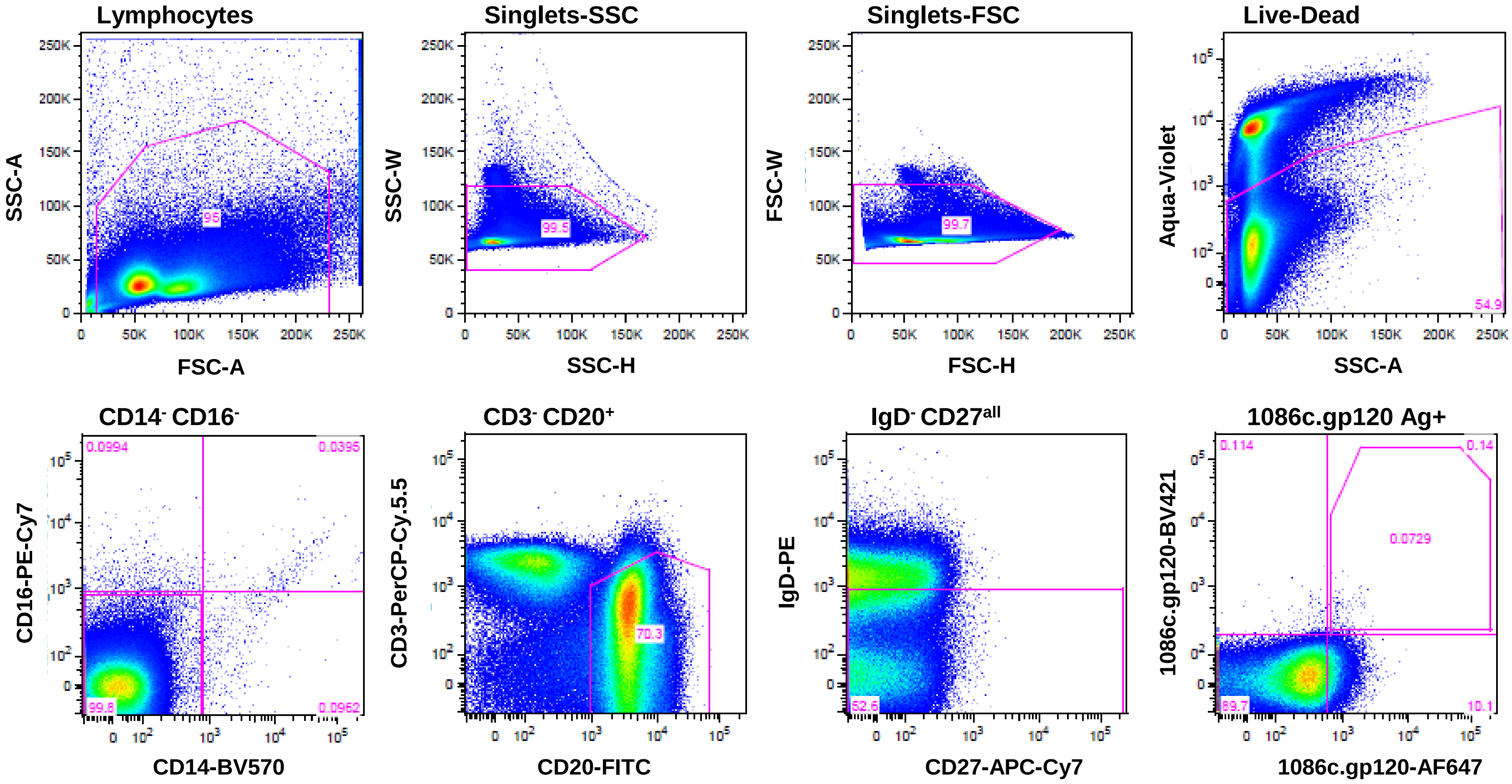

S1 Fig

D. Co-administration group, animal 45038, PBMC (W12), gating strategy: lymphocytes → Singlets (SSC or FSC) → Live-dead → CD14-CD16- → CD3-CD20+ → IgD-CD27all → 1086c.gp120 double positive

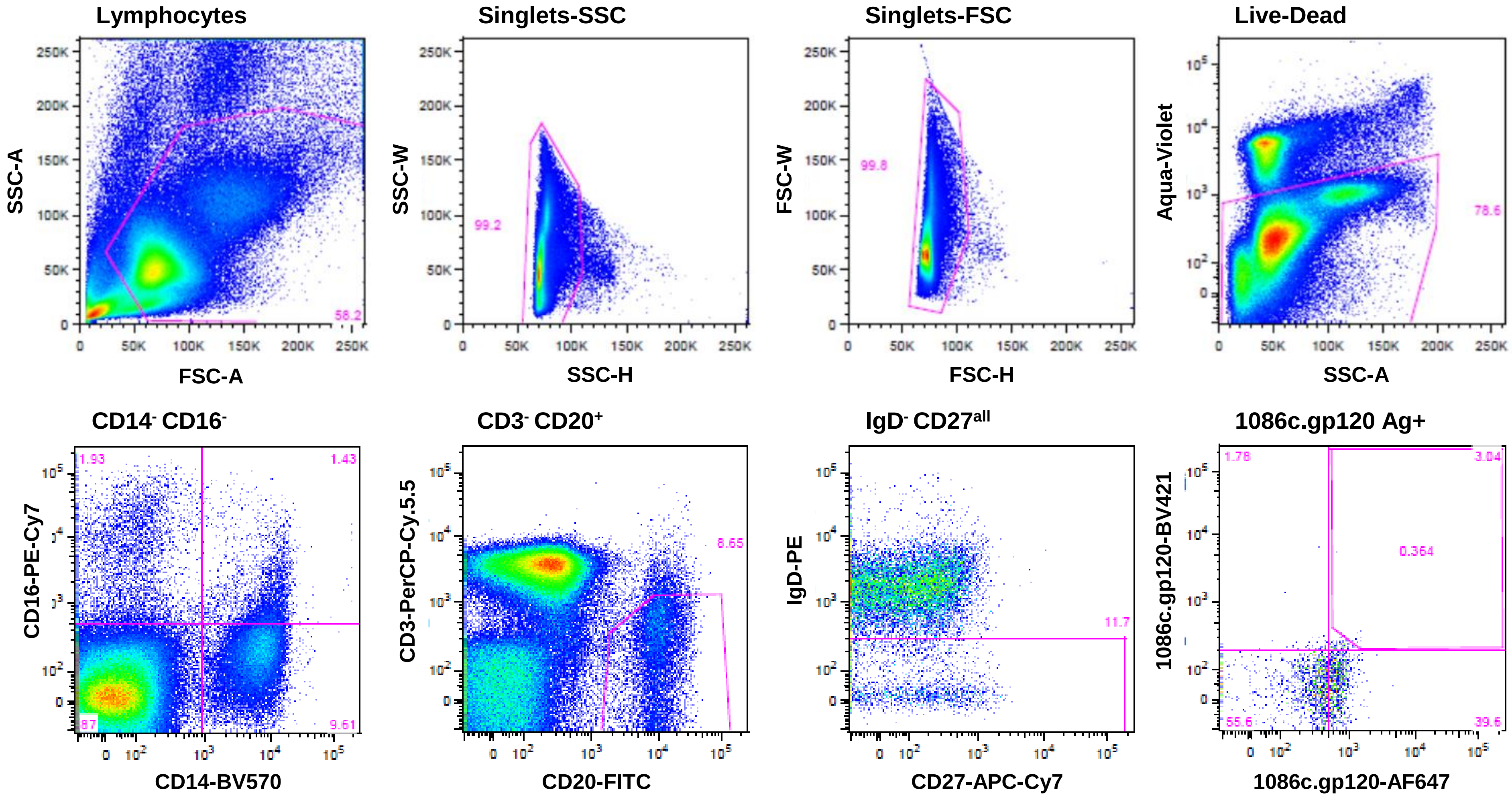

S1 Fig

E. Co-administration group, animal 45083, Spleen (necropsy), gating strategy: lymphocytes → Singlets (SSC or FSC) → Live-dead → CD14-CD16- → CD3-CD20+ → IgD-CD27all → 1086c.gp120 double positive

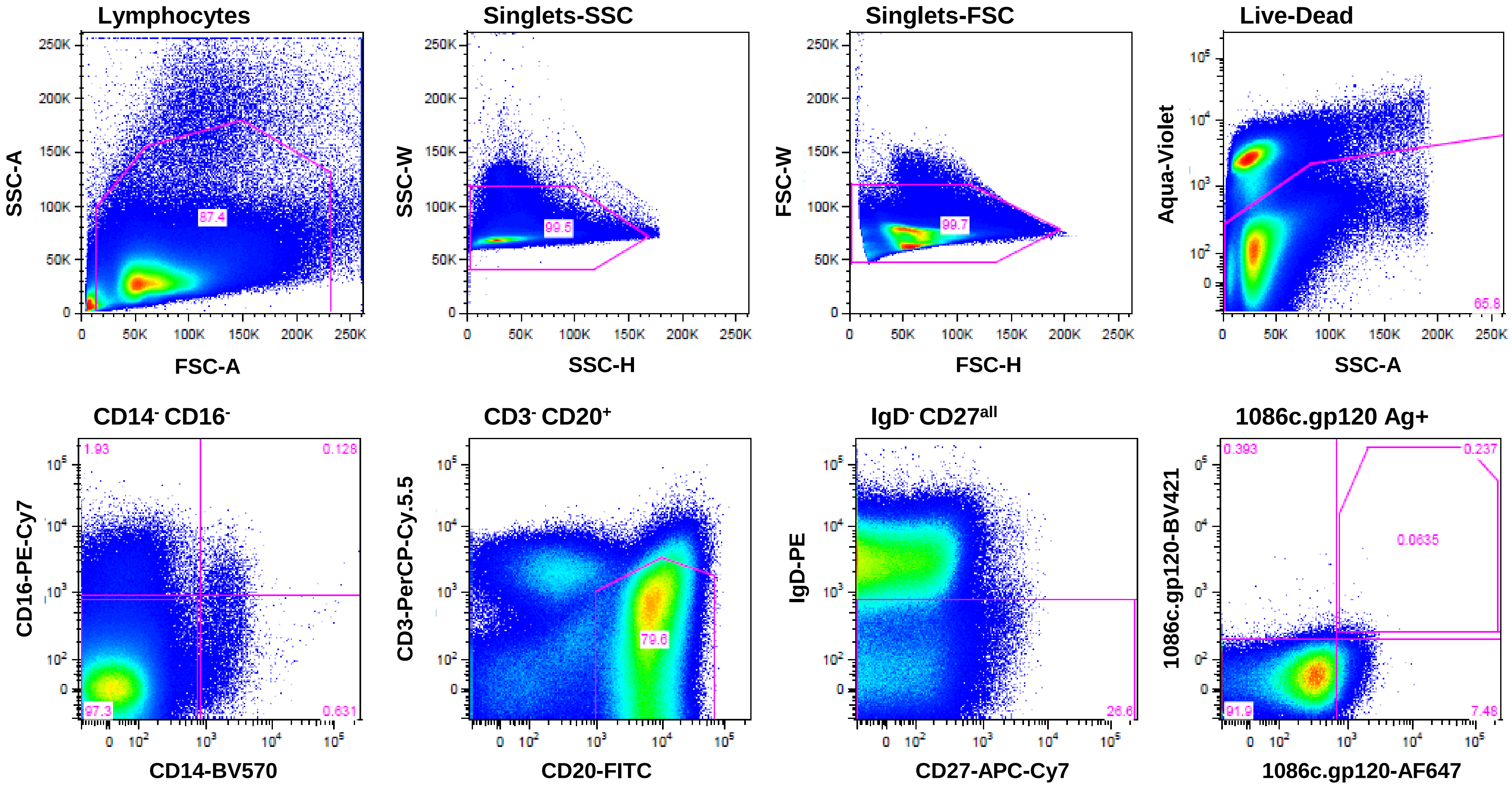

S1 Fig

F. Co-administration group, animal 45091, Axillary LN (necropsy), gating strategy: lymphocytes → Singlets (SSC or FSC) → Live-dead → CD14-CD16- → CD3-CD20+ → IgD-CD27all → 1086c.gp120 double positive

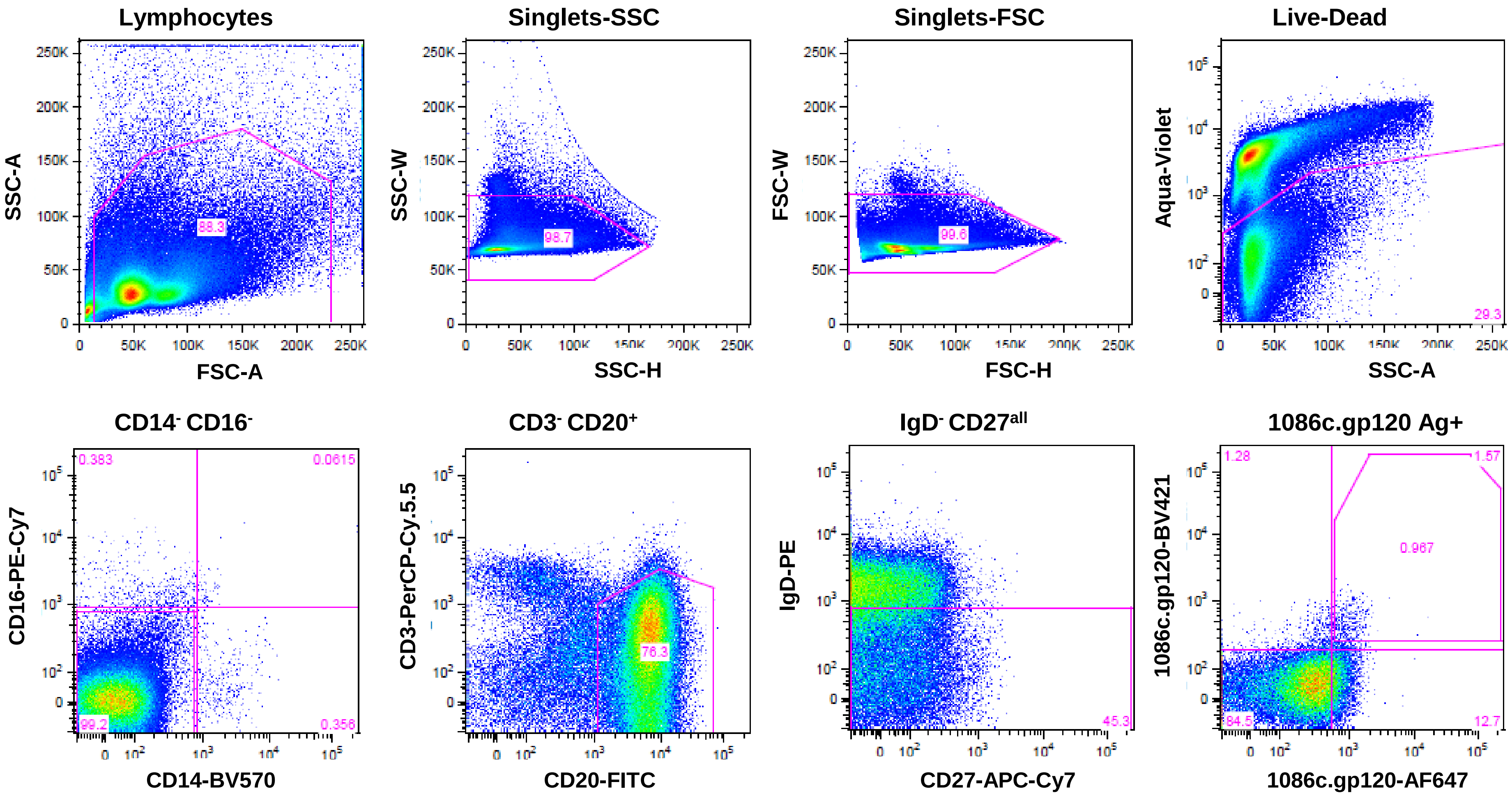

S1 Fig

G. Extended Interval group, animal 45435, Spleen (necropsy), representative data of 3 independent sort, gating strategy: lymphocytes → Singlets (SSC or FSC) → Live-dead → CD14-CD16- → CD3-CD20+ → IgD-CD27all → 1086c.gp120 double positive

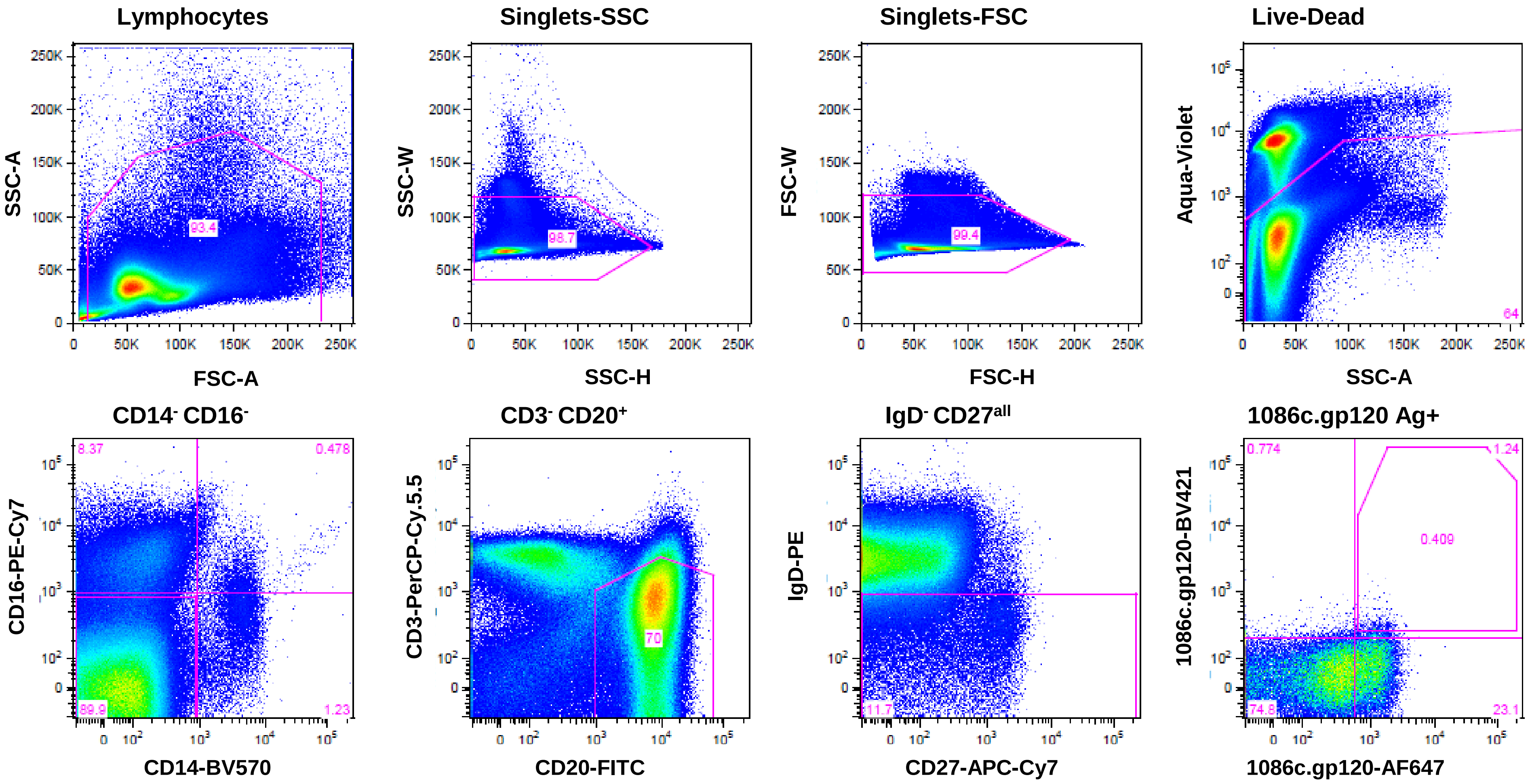

S1 Fig

H. Extended Interval group, animal 45441, Oral LN (submandibular, necropsy), representative data of 4 independent sort, gating strategy: lymphocytes → Singlets (SSC or FSC) → Live-dead → CD14-CD16- → CD3-CD20+ → IgD-CD27all → 1086c.gp120 double positive

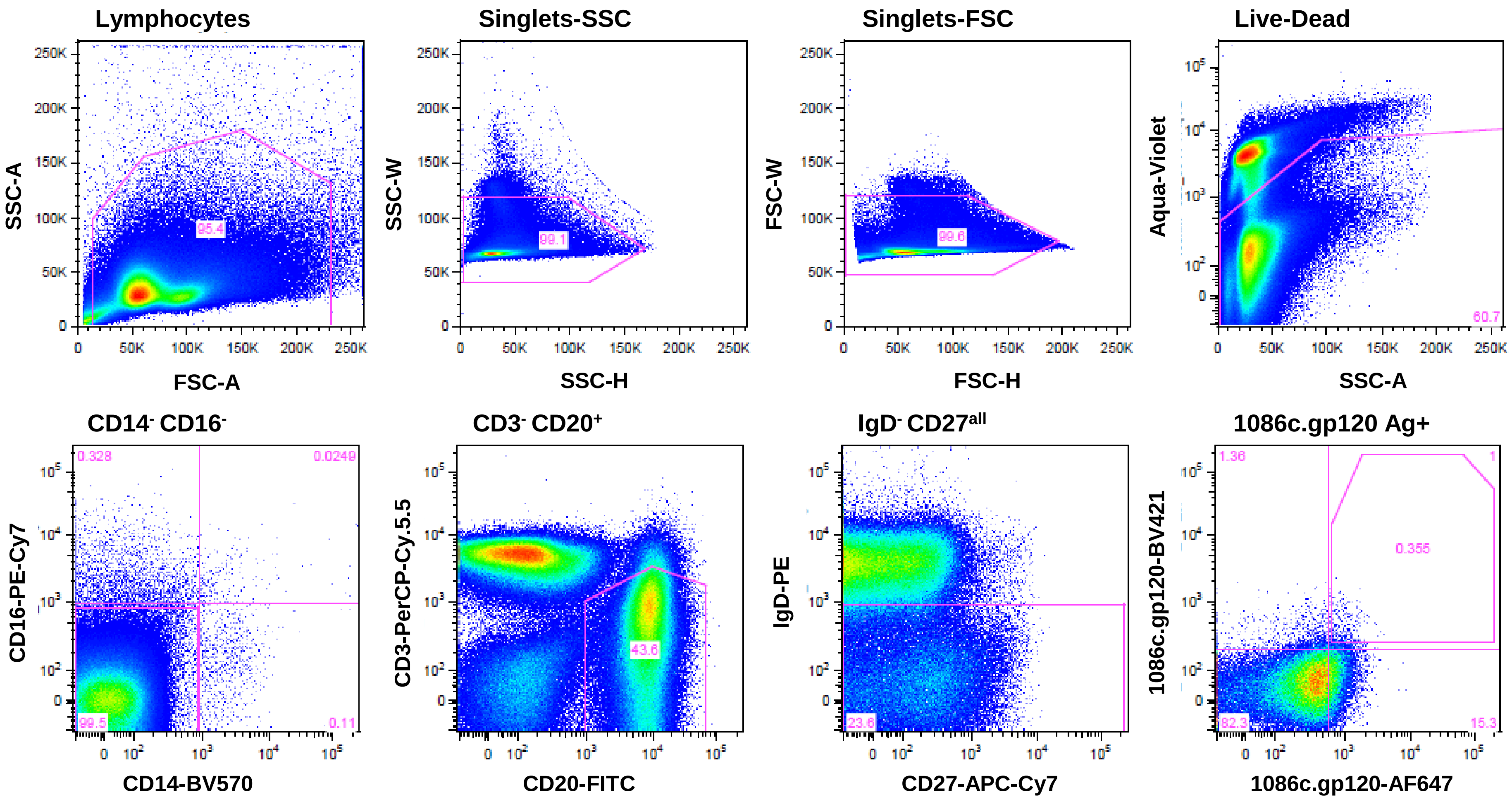

S1 Fig

I. Extended Interval group, animal 45448, Colon (necropsy), representative data of 3 independent sort, gating strategy: lymphocytes → Singlets (SSC or FSC) → Live-dead → CD14-CD16- → CD3-CD20+ → IgD-CD27all → 1086c.gp120 double positive

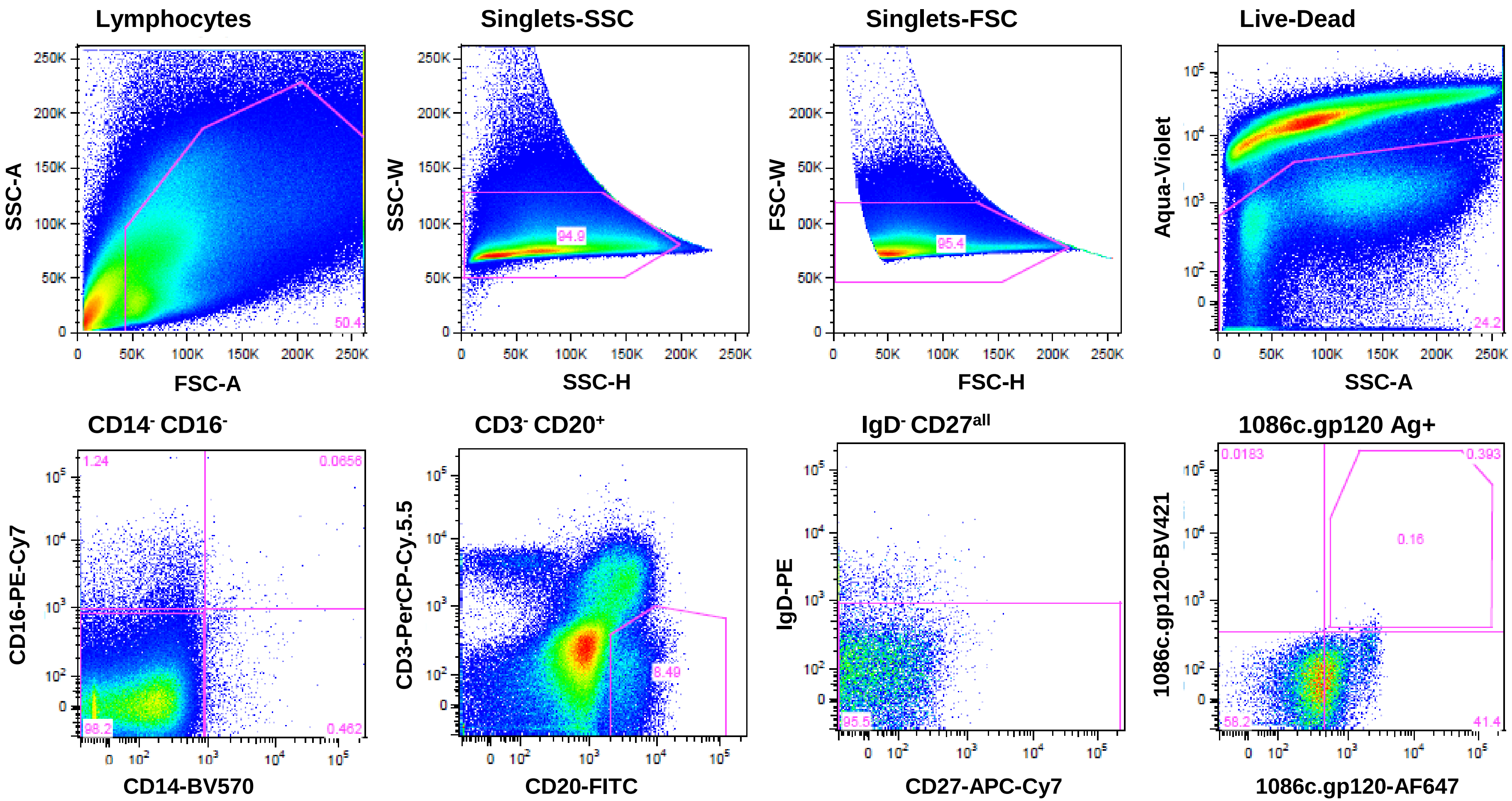

S1 Fig

J. 3M-052-SE group, animal 45838, PBMC (W12), gating strategy: lymphocytes → Singlets (SSC or FSC) → Live-dead → CD14-CD16- → CD3-CD20+ → IgD-CD27all → 1086c.gp120 double positive

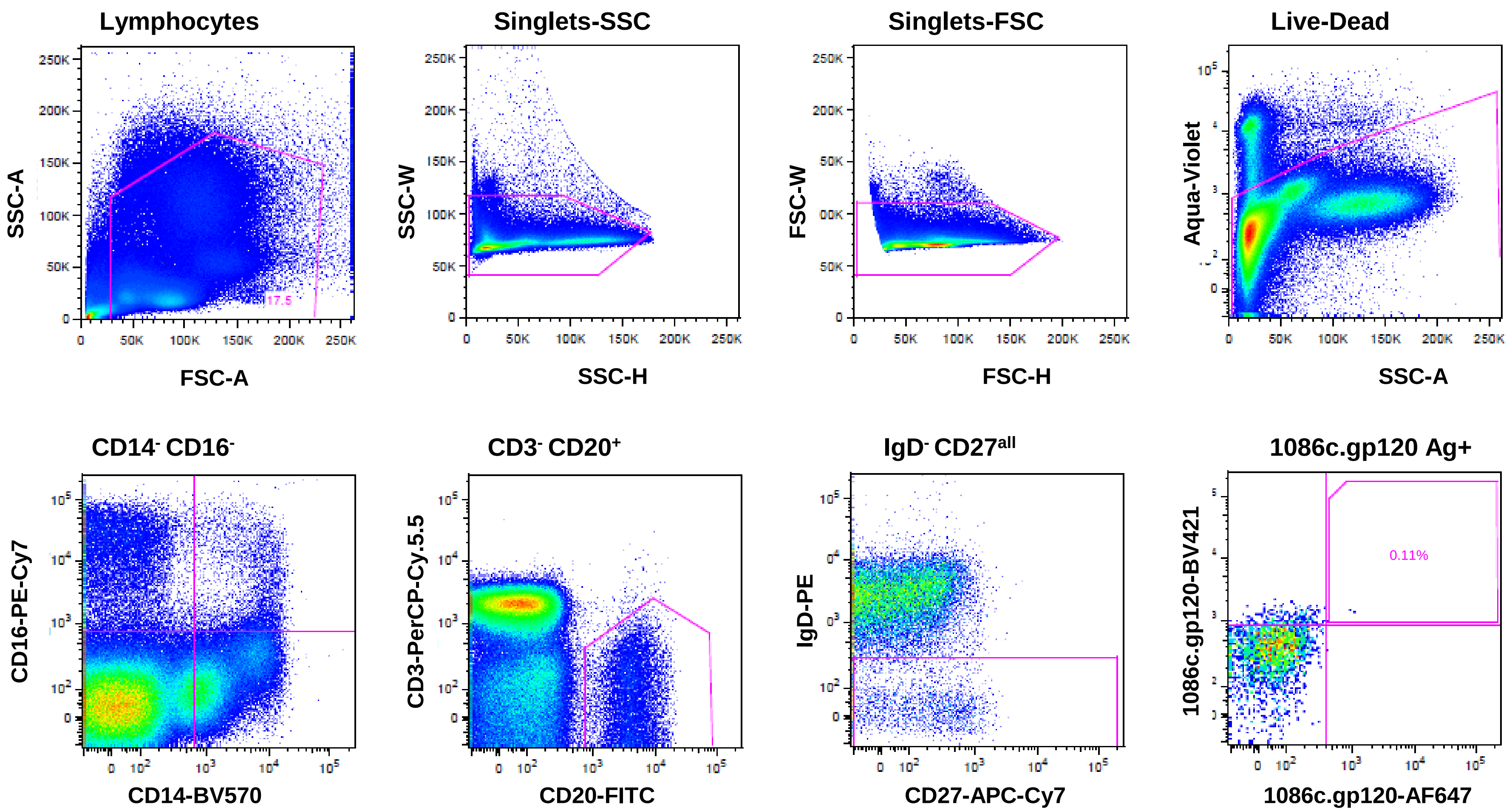

S1 Fig 1A-J. Representative flow cytometry analyses for single cell HIV Env-specific memory B cell sorting from PBMC and tissues of infants by vaccination groups. Percentage of memory B cells (CD20+CD27+IgD-), which were reactive to HIV 1086c gp120 K160N tagged with two colors-BV421 and AF647 (double positive cells) indicated in the upper right quadrant. Gates were drawn based on isotype controls and fluorescent minus one (FMO) controls.
