# Supplementary material for: Different adjuvanted pediatric HIV envelope vaccines induced distinct plasma antibody responses despite similar B cell receptor repertoires in infant rhesus macaques
Source: PLoS One. 2021 Dec 31;16(12):e0256885. doi: 10.1371/journal.pone.0256885 (PMC8719683; doi:10.1371/journal.pone.0256885)
Supplement: S2 Fig — (A) Cross clade HIV gp120-specific IgG responses at peak immunogenicity by luminex assay (BAMA). (B) Variable and conserved epitopes-specific IgG responses at peak immunogenicity by BAMA. (C) The 3M-052 group antibody responses had higher avidity strength against most of the tested antigens. Avidity 1/k off (the inverse of the dissociation rate) was plotted as a measure of the strength of binding and avidity scores which take into consideration the magnitude are also shown. Statistical analyses were performed with GraphPad Prism, * denoted significant p-values of <0.05 by a non-parametric Mann-Whitney test. (PDF) [file pone.0256885.s002.pdf]

S2 Fig

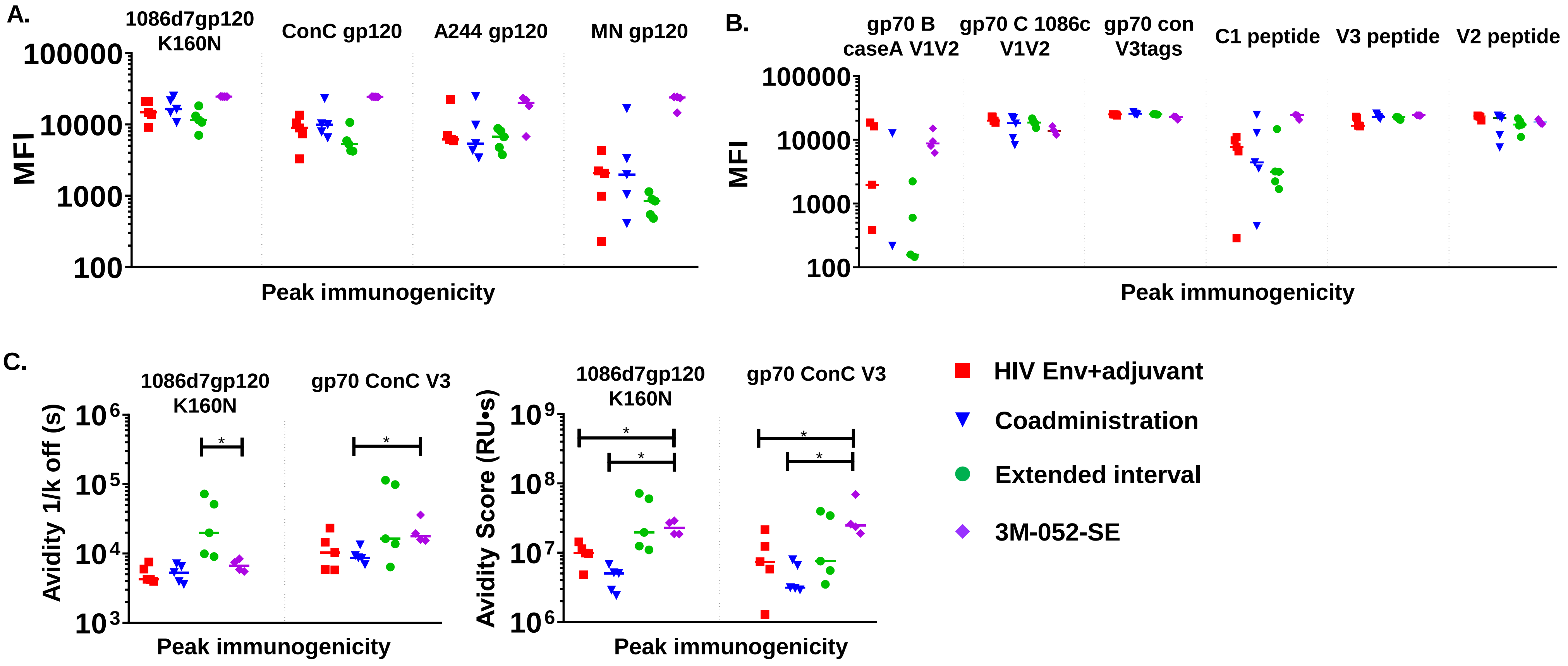

S2 Fig. Characterization of vaccination-induced plasma envelope IgG responses in infant rhesus macaques. (A) Cross clade HIV gp120-specific IgG responses at peak immunogenicity by luminex assay (BAMA). (B) Variable and conserved epitopes-specific IgG responses at peak immunogenicity by BAMA. (C) The 3M-052 group antibody responses had higher avidity strength against most of the tested antigens. Avidity 1/k off (the inverse of the dissociation rate) was plotted as a measure of the strength of binding and avidity scores which take into consideration the magnitude are also shown. Statistical analyses were performed with GraphPad Prism, * denoted significant p-values of <0.05 by a non-parametric Mann-Whitney test.
