# Supplementary material for: Different adjuvanted pediatric HIV envelope vaccines induced distinct plasma antibody responses despite similar B cell receptor repertoires in infant rhesus macaques
Source: PLoS One. 2021 Dec 31;16(12):e0256885. doi: 10.1371/journal.pone.0256885 (PMC8719683; doi:10.1371/journal.pone.0256885)
Supplement: S3 Fig — Initial analysis with human immunoglobulin (Ig) database indicated a total of 39 heavy- and light-chain pairs isolated from antigen-specific memory B cells across different vaccine groups. Epitope specificity, VH gene family usage, and isotype distribution of identified functional heavy- and light-chain pairs were similar across vaccine groups. Epitope specificity, VH gene family usage, and isotype distribution of identified functional heavy and light chains are displayed in concentric circles. The number of mAbs per group is displayed in the center. (PDF) [file pone.0256885.s003.pdf]

S3 Fig

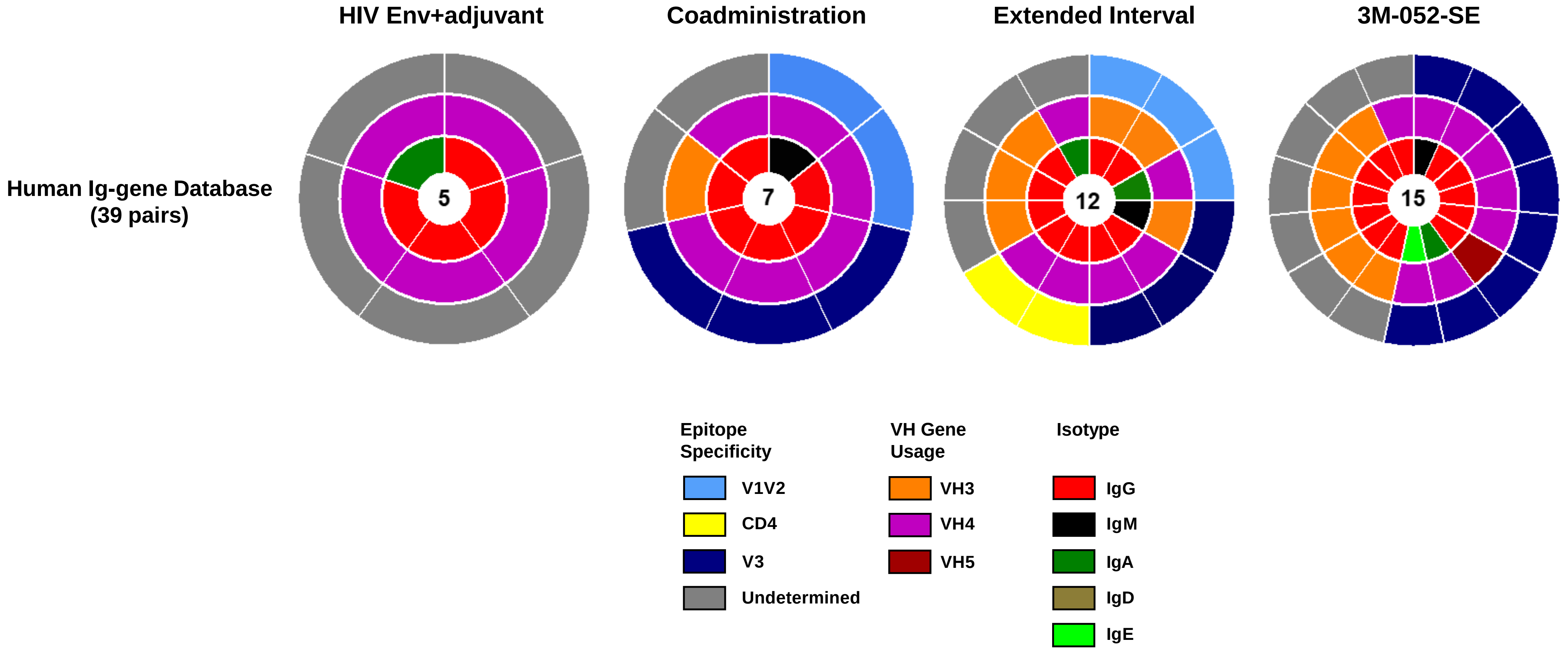

S3 Fig. Analyses of epitope specificity and immunogenetic characteristics of the Env-specific functional heavy- and light-chains of 39 vaccine-elicited mAbs in infants using human Ig-gene database. Initial analysis with human immunoglobulin (Ig) database indicated a total of 39 heavy- and light-chain pairs isolated from antigen-specific memory B cells across different vaccine groups. Epitope specificity, VH gene family usage, and isotype distribution of identified functional heavy- and light-chain pairs were similar across vaccine groups. Epitope specificity, VH gene family usage, and isotype distribution of identified functional heavy and light chains are displayed in concentric circles. The number of mAbs per group is displayed in the center.
