# Supplementary material for: Different adjuvanted pediatric HIV envelope vaccines induced distinct plasma antibody responses despite similar B cell receptor repertoires in infant rhesus macaques
Source: PLoS One. 2021 Dec 31;16(12):e0256885. doi: 10.1371/journal.pone.0256885 (PMC8719683; doi:10.1371/journal.pone.0256885)
Supplement: S4 Fig — Analysis of percent somatic hypermutation frequency and HCDR3 lengths for Env-reactive heavy and light chains pairs (39 mAb pairs) from infant antigen-specific B cells based on human immunoglobulin (Ig) sequence database. Horizontal lines indicated median values of individual groups. Corresponding functional heavy and light chains isolated from individual infants are denoted by symbols. (PDF) [file pone.0256885.s004.pdf]

S4 Fig

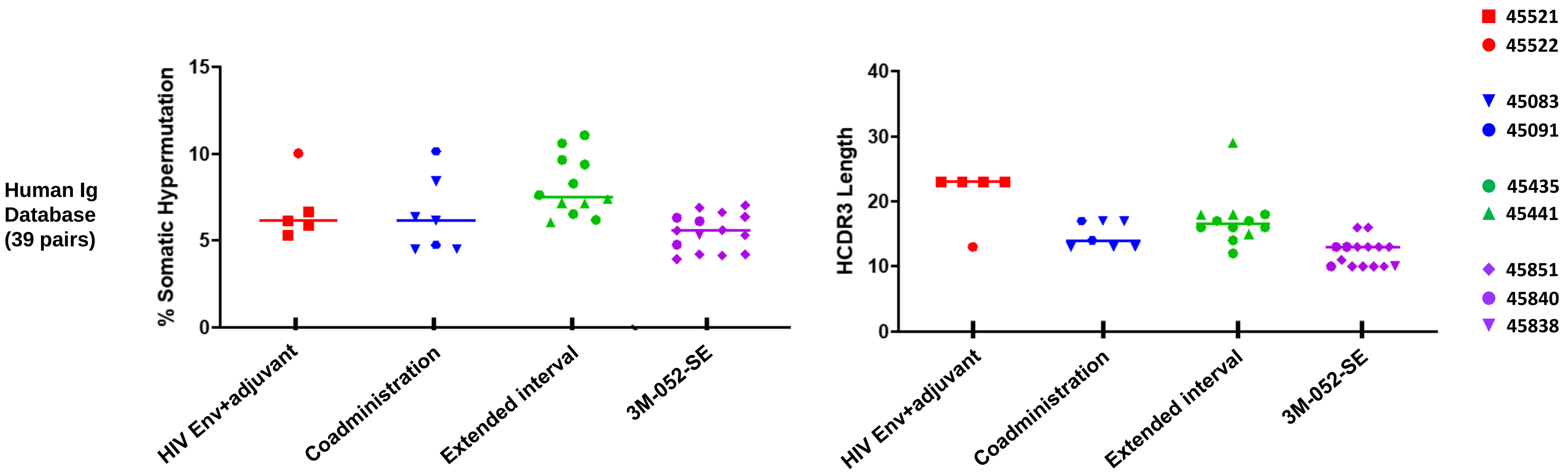

S4 Fig. Frequency of somatic hypermutation and heavy chain complementarity-determining region 3 (HCDR3) length of vaccine-elicited Env-reactive functional heavy- and light-chains identified using rhesus Ig sequence database. Analysis of percent somatic hypermutation frequency and HCDR3 lengths for Env-reactive heavy and light chains pairs (39 mAb pairs) from infant antigen-specific B cells based on human immunoglobulin (Ig) sequence database. Horizontal lines indicated median values of individual groups. Corresponding functional heavy and light chains isolated from individual infants are denoted by symbols.
