# Supplementary material for: Different adjuvanted pediatric HIV envelope vaccines induced distinct plasma antibody responses despite similar B cell receptor repertoires in infant rhesus macaques
Source: PLoS One. 2021 Dec 31;16(12):e0256885. doi: 10.1371/journal.pone.0256885 (PMC8719683; doi:10.1371/journal.pone.0256885)
Supplement: S1 Table — A total of 39 pairs of potentially Env-reactive mAbs were isolated from the four vaccination groups across several anatomic compartments. Frequency of gene usage, percent somatic hypermutation, and complementarity-region 3 (CDR3) length are displayed for the heavy and light chains for each mAb along with the isotype and epitope specificity. (PDF) [file pone.0256885.s005.pdf]

S1 Table. Immunogenetic characteristics of isolated envelope (Env)-reactive mAbs of Env-vaccinated infant monkeys based on human immunoglobulin database analysis. A total of 39 pairs of potentially Env-reactive mAbs were isolated from the four vaccination groups across several anatomic compartments. Frequency of gene usage, percent somatic hypermutation, and complementarity-region 3 (CDR3) length are displayed for the heavy and light chains for each mAb along with the isotype and epitope specificity.

Animal ID	Group	Tissue	IgH ID	V_H gene	D_H gene	J_H gene	HC % SHM	HC CDR3 length	Ig Isotype	IgL ID	V_L/V_K gene	J_L/J_K gene	Specificity
45521	HIV Env+adjuvant	Spleen	H020465	4~4*07	2~OF15*2/inv	3*01	5.31	23	IgG	K020382	1~33*01	2*03	Undetermined
45521	HIV Env+adjuvant	Retropharyngeal LN	H914640	4~4*07	3~3*01	3*01	6.14	23	IgA	K907482	1~33*01	2*03	Undetermined
45521	HIV Env+adjuvant	Retropharyngeal LN	H914648	4~4*07	3~3*01	3*01	6.65	23	IgG	K907485	1~33*01	2*03	Undetermined
45521	HIV Env+adjuvant	Retropharyngeal LN	H914649	4~4*07	5~12*01	3*01	5.88	23	IgG	K907486	1~33*01	2*03	Undetermined
45522	HIV Env+adjuvant	Spleen	H020414	4~61*03	6~13*01	4*02	10.03	13	IgG	L020264	1~51*02	3*02	Undetermined
45083	Coadministration	Spleen	H020400	4~59*01	6~19*01	4*02	6.17	13	IgM	L020253	1~40*01,02	2*01	V1V2
45083	Coadministration	Mediastinal LN	H020405	4~59*01,02	3~16*01,02	4*02	4.52	17	IgG	K020331	1D~16*01	2*03,04	V1V2
45083	Coadministration	Mediastinal LN	H020405	4~59*01,02	3~16*01,02	4*02	4.52	17	IgG	L020258	2~23*02	1*01	V3
45083	Coadministration	Mediastinal LN	H020407	4~b*02	5~12*01	4*02	8.41	13	IgG	L020260	2~8*01	1*01	V3
45083	Coadministration	Mediastinal LN	H020408	4~39*06	1~26*01	4*02	6.38	13	IgG	L020260	2~8*01	1*01	Undetermined
45091	Coadministration	Axillary LN	H020381	3~73*01,02	2~2*02/inv	6*02	10.15	14	IgG	L020241	11~55*01	2*01	Undetermined
45091	Coadministration	Axillary LN	H020387	4~59*01	4~4*01	4*02	4.74	17	IgG	K020324	1~39*01	4*01	V3
45435	Extended Interval	Mediastinal LN	H020422	4~59*01	3~3*01,02	3*01,02	6.19	18	IgA	K020341	1~13*02	4*01	V1V2
45435	Extended Interval	Mediastinal LN	H020425	4~59*01,02	3~OR15*3	5*01,02	7.63	16	IgG	K020344	1~33*01	2*03	CD4 binding site
45435	Extended Interval	Mediastinal LN	H020426	3~72*01	1~OR15*1	6*02	8.30	14	IgM	L020282	11~55*01	2*01	V3
45435	Extended Interval	Mediastinal LN	H020430	3~11*01	1~7*01/inv	5*01	10.61	17	IgG	K020348	1/OR2~0*01	2*03	V1V2
45435	Extended Interval	Mediastinal LN	H020431	3~64*02	4~17*01	5*01	9.40	12	IgG	K020349	3~11*01	1*01	V1V2
45435	Extended Interval	Mediastinal LN	H020445	4~4*02	4~4*01	4*02	6.53	16	IgG	K020359	1D~16*01	2*03	V3
45435	Extended Interval	Mediastinal LN	H020420	4~61*05	6~13*01	4*02	11.08	16	IgG	L020280	5~39*01	2*01	V3
45435	Extended Interval	Spleen	H020461	3~73*01,02	2~15*01	4*02	9.65	17	IgG	K020380	1~12*01,02	2*03	Undetermined
45441	Extended Interval	Spleen	H020449	3~21*01,02	1~IR1*01	1*01	6.05	29	IgG	K020361	1D~16*01	1*01	Undetermined
45441	Extended Interval	Spleen	H020452	4~59*01	3~3*01	4*02	7.18	18	IgG	L020292	1~51*02	7*01	CD4 binding site
45441	Extended Interval	Spleen	H020450	3~21*01,02	3~22*01	1*01	7.14	15	IgG	L020290	3~21*01	6*01	Undetermined
45441	Extended Interval	Submental LN	H914598	4~59*01	3~3*01	6*02	7.41	18	IgA	K907464	2~28*01	1*01	Undetermined
45838	3M-052-SE	PBMC	H020493	3~43*02	6~13*01	4*02	5.31	10	IgG	L020321	3~19*01	2*01	Undetermined
45840	3M-052-SE	PBMC	H020481	4~61*01,08	3~3*01,02	4*02	6.32	13	IgG	K020401	2~30*01	1*01	V3
45840	3M-052-SE	PBMC	H020485	4~39*06	3~3*01,02	4*02	6.12	13	IgG	K020402	2~40*01	1*01	V3
45840	3M-052-SE	PBMC	H020488	3~43*02	6~13*01	4*02	4.77	10	IgG	K020402	2~40*01	1*01	Undetermined
45851	3M-052-SE	PBMC	H020495	3~15*08	0~IR*01C	3*01	7.03	11	IgG	K020407	2~40*01	1*01	Undetermined
45851	3M-052-SE	PBMC	H020494	4~39*06	3~3*01,02	4*02	6.89	13	IgG	K020409	2~30*01	1*01	Undetermined
45851	3M-052-SE	PBMC	H020500	4~59*01	6~13*01	1*01	4.21	10	IgA	L020324	5~48*01	1*01	V3
45851	3M-052-SE	PBMC	H020498	5~51*01	3~10*01	5*01	4.15	13	IgG	L020327	1~50*01	2*01	V3
45851	3M-052-SE	PBMC	H020499	3~43*02	2~8*02	4*02	5.61	16	IgG	L020326	6~57*01	7*01	Undetermined
45851	3M-052-SE	PBMC	H020505	4~59*01	1~1*01	1*01	4.21	10	IgM	L020329	6~57*01	7*01	V3
45851	3M-052-SE	PBMC	H020501	4~39*06	3~3*01,02	4*02	6.63	13	IgG	L020328	5~48*01	1*01	V3
45851	3M-052-SE	PBMC	H020504	3~43*02	6~13*01	4*02	5.31	10	IgG	L020329	6~57*01	7*01	Undetermined
45851	3M-052-SE	PBMC	H020506	4~39*06	3~3*01,02	1*01	6.38	13	IgE	L020333	6~57*01	7*01	V3
45851	3M-052-SE	PBMC	H020496	3~43*02	2~8*02	4*02	5.57	16	IgG	K020407	2~40*01	1*01	Undetermined
45851	3M-052-SE	PBMC	H020497	4~59*01	1~1*01	1*01	3.95	10	IgG	K020409	2~30*01	1*01	V3
