# Supplementary material for: Different adjuvanted pediatric HIV envelope vaccines induced distinct plasma antibody responses despite similar B cell receptor repertoires in infant rhesus macaques
Source: PLoS One. 2021 Dec 31;16(12):e0256885. doi: 10.1371/journal.pone.0256885 (PMC8719683; doi:10.1371/journal.pone.0256885)
Supplement: S1 Data — (PDF) [file pone.0256885.s006.pdf]

S1 Fig

A. HIV Env+adjuvant group, animal 45519, PBMC (W10), gating strategy: lymphocytes → Singlets (SSC or FSC) → Live-dead → CD14-CD16- → CD3-CD20+ → IgD-CD27all → 1086c.gp120 double positive

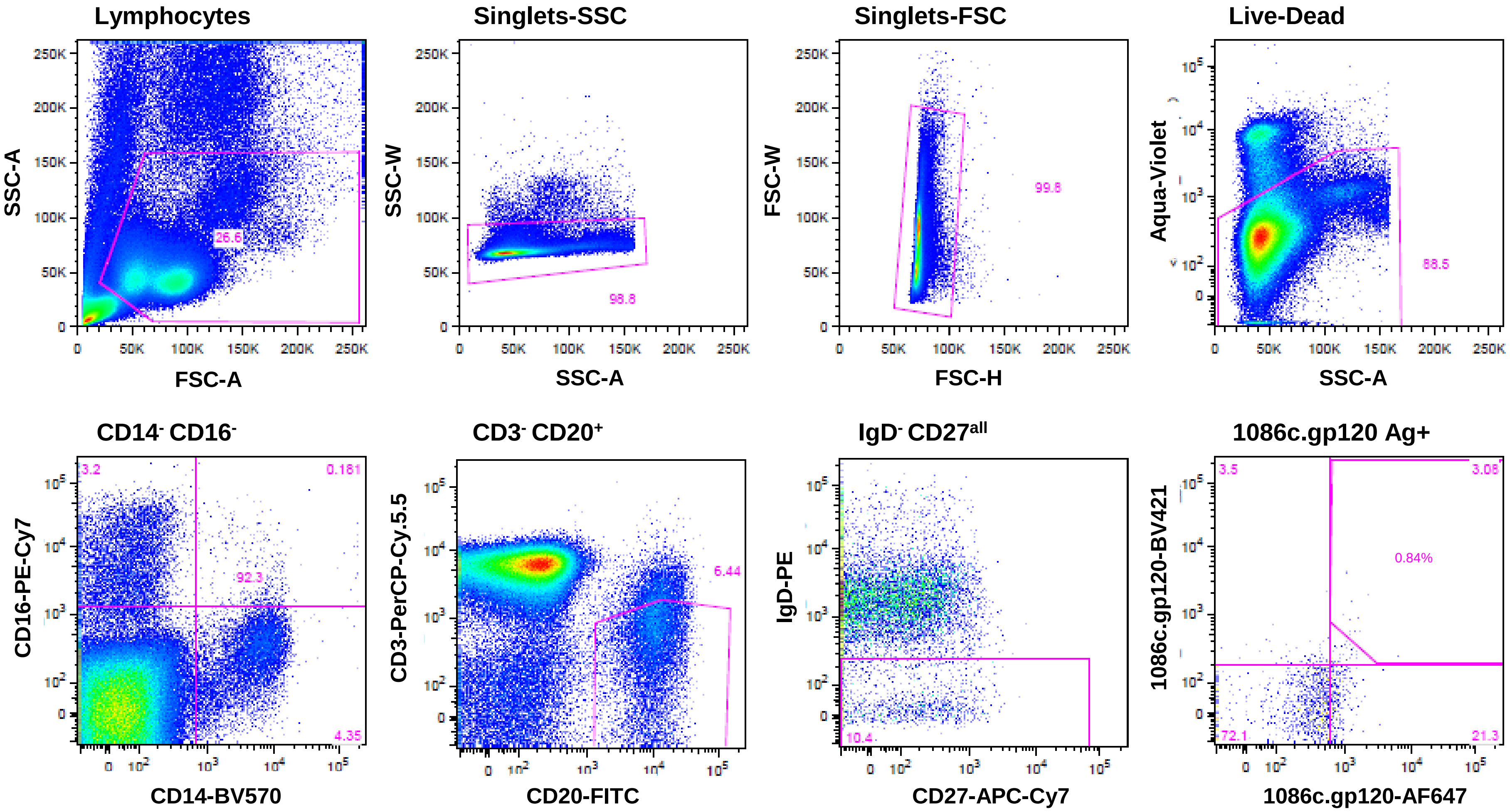

S1 Fig

B. HIV Env+adjuvant group, animal 45521, Spleen (necropsy), representative data of 2 independent sort, gating strategy: lymphocytes → Singlets (SSC or FSC) → Live-dead → CD14-CD16- → CD3-CD20+ → IgD-CD27all → 1086c.gp120 double positive

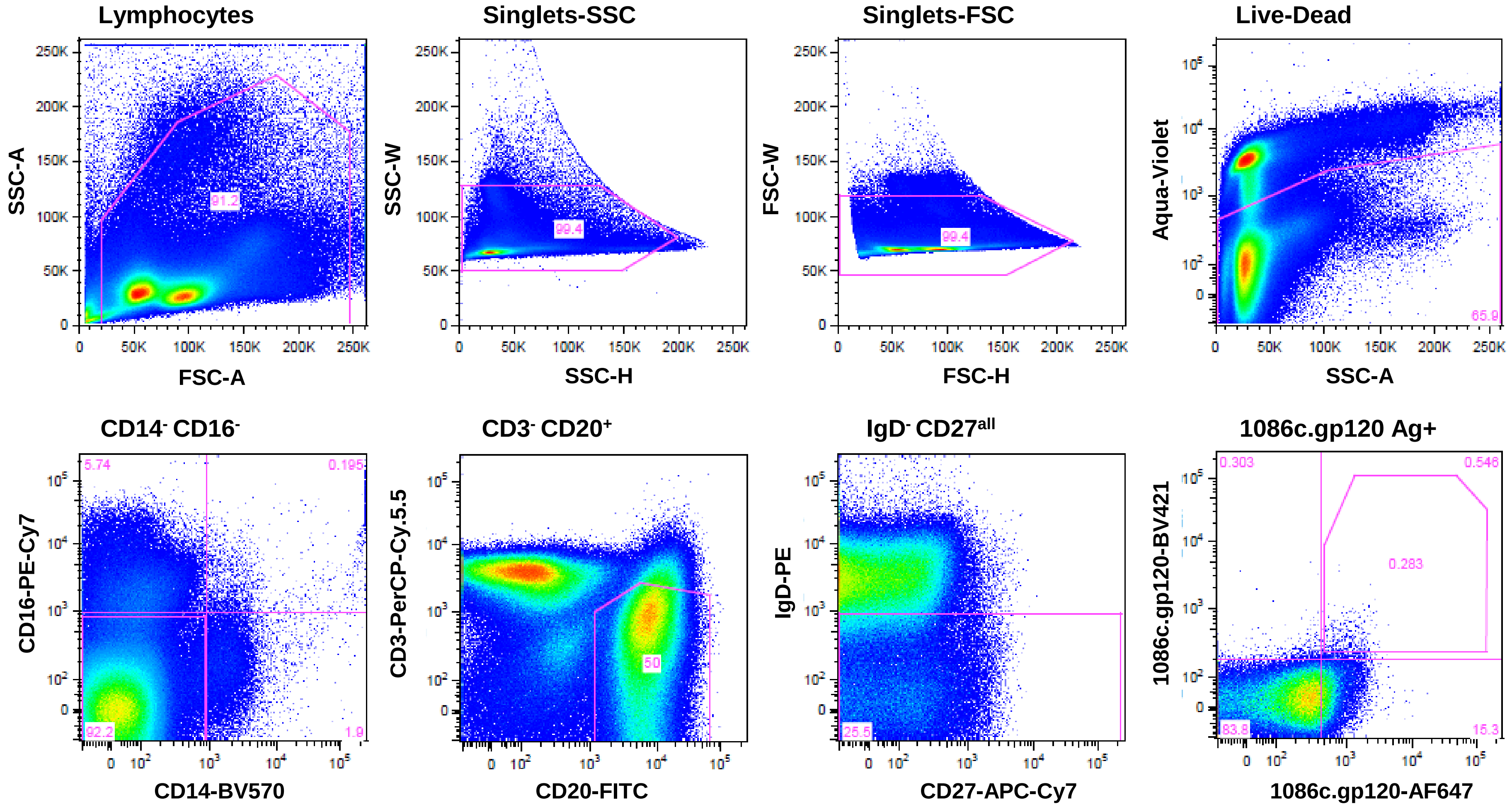

S1 Fig

C. HIV Env+adjuvant group, animal 45522, Oral LN (retropharengeal, necropsy), representative data of 3 independent sort, gating strategy: lymphocytes → Singlets (SSC or FSC) → Live-dead → CD14-CD16- → CD3-CD20+ → IgD-CD27all → 1086c.gp120 double positive

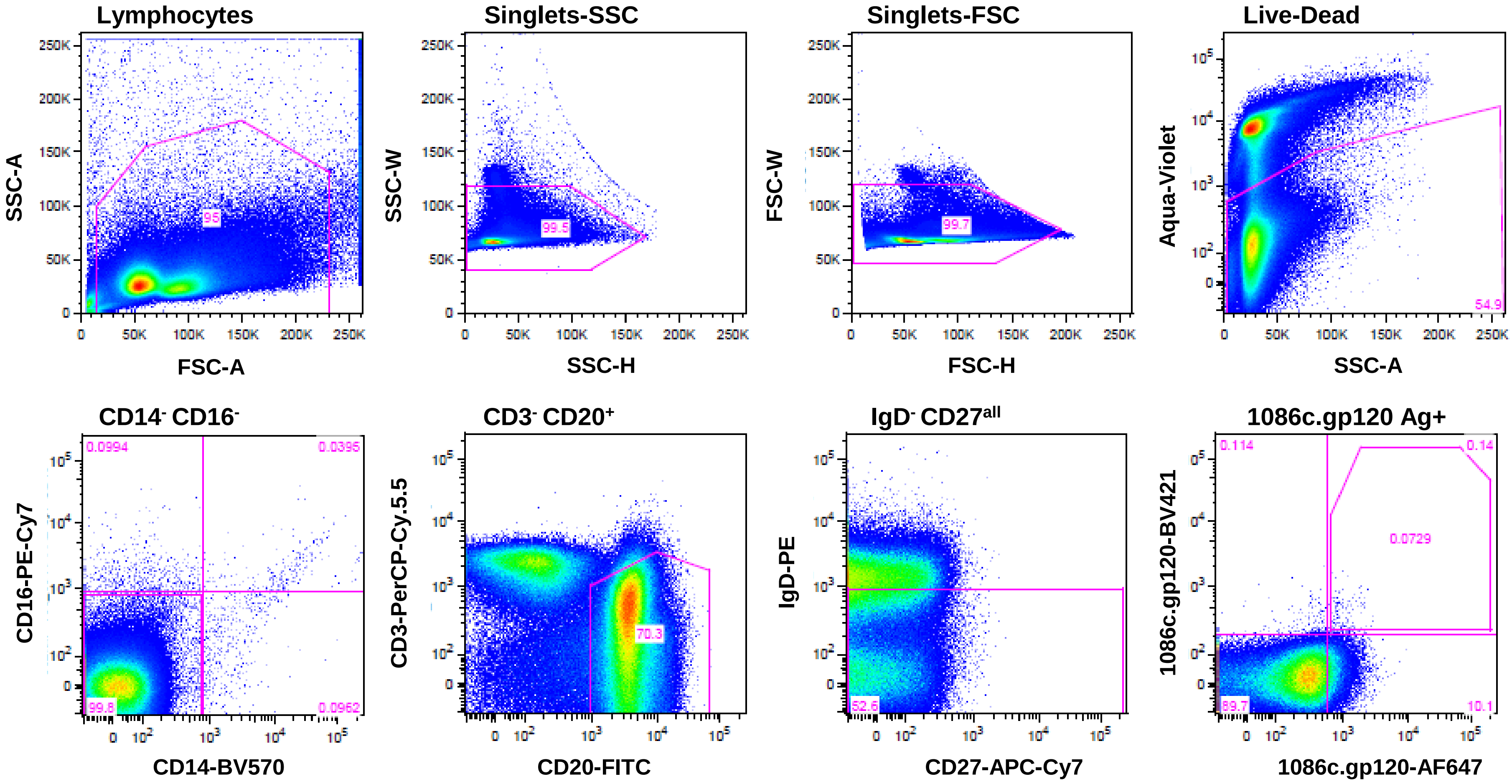

S1 Fig

D. Co-administration group, animal 45038, PBMC (W12), gating strategy: lymphocytes → Singlets (SSC or FSC) → Live-dead → CD14-CD16- → CD3-CD20+ → IgD-CD27all → 1086c.gp120 double positive

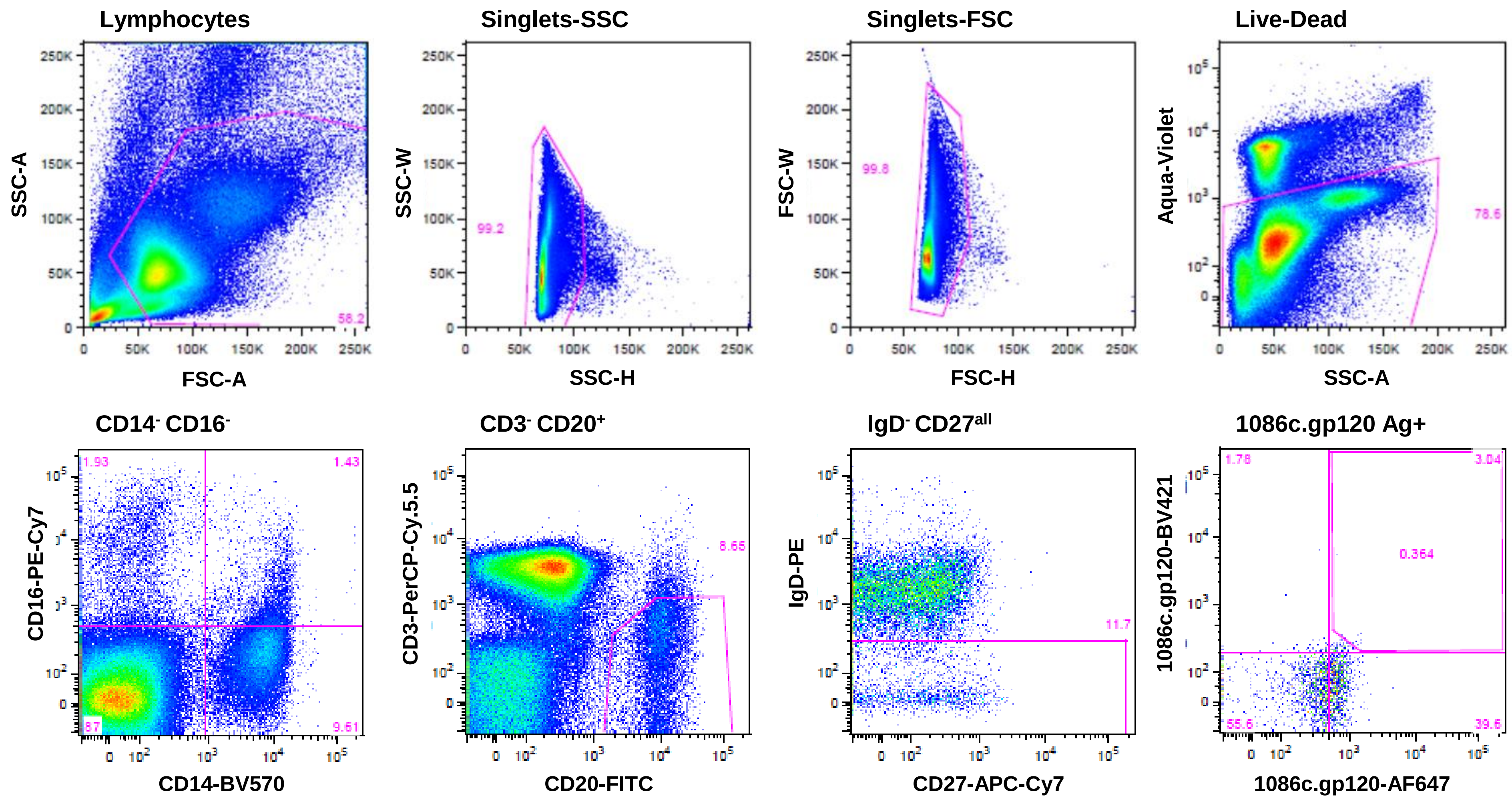

S1 Fig

E. Co-administration group, animal 45083, Spleen (necropsy), gating strategy: lymphocytes → Singlets (SSC or FSC) → Live-dead → CD14-CD16- → CD3-CD20+ → IgD-CD27all → 1086c.gp120 double positive

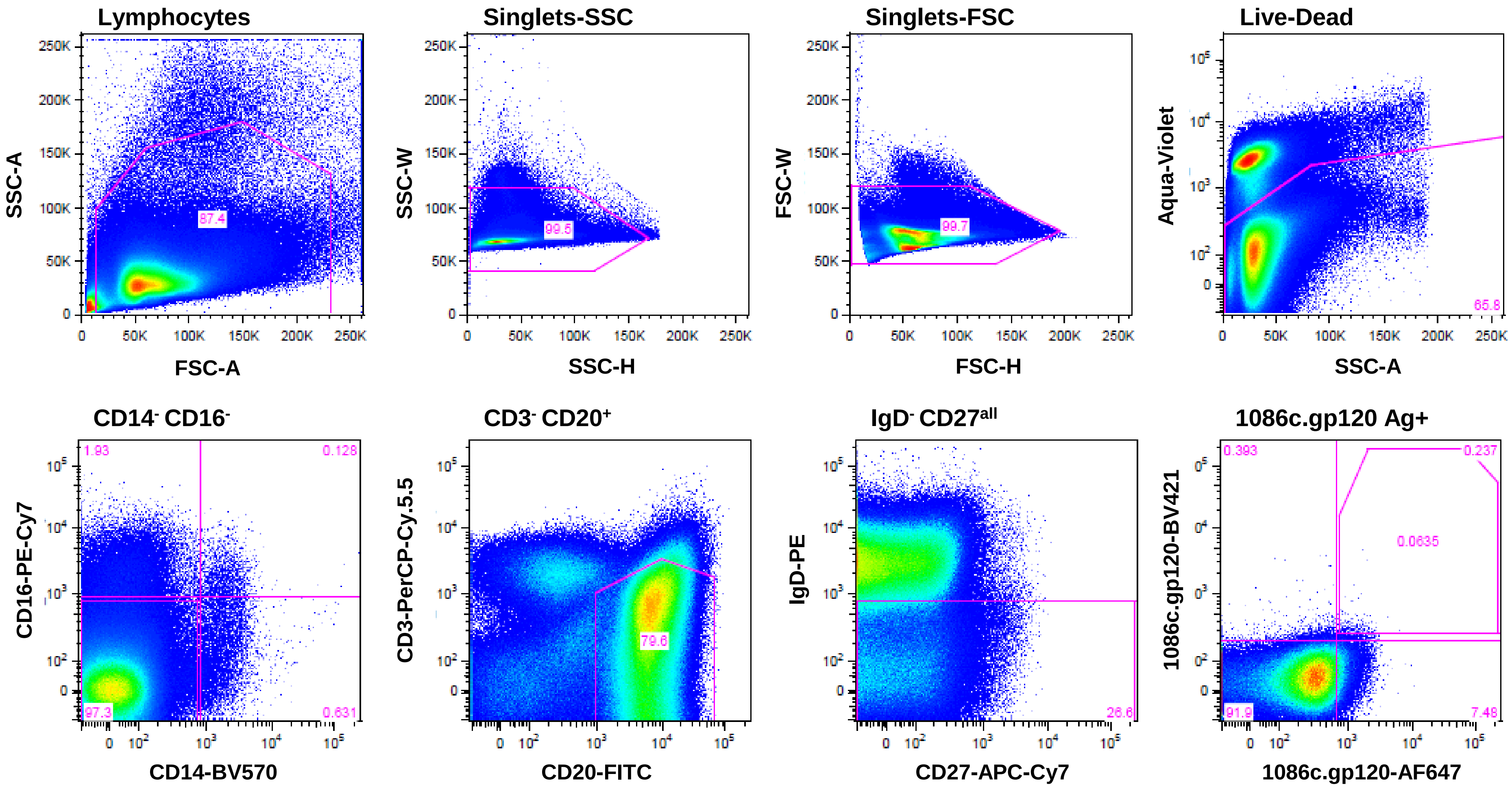

S1 Fig

F. Co-administration group, animal 45091, Axillary LN (necropsy), gating strategy: lymphocytes → Singlets (SSC or FSC) → Live-dead → CD14-CD16- → CD3-CD20+ → IgD-CD27all → 1086c.gp120 double positive

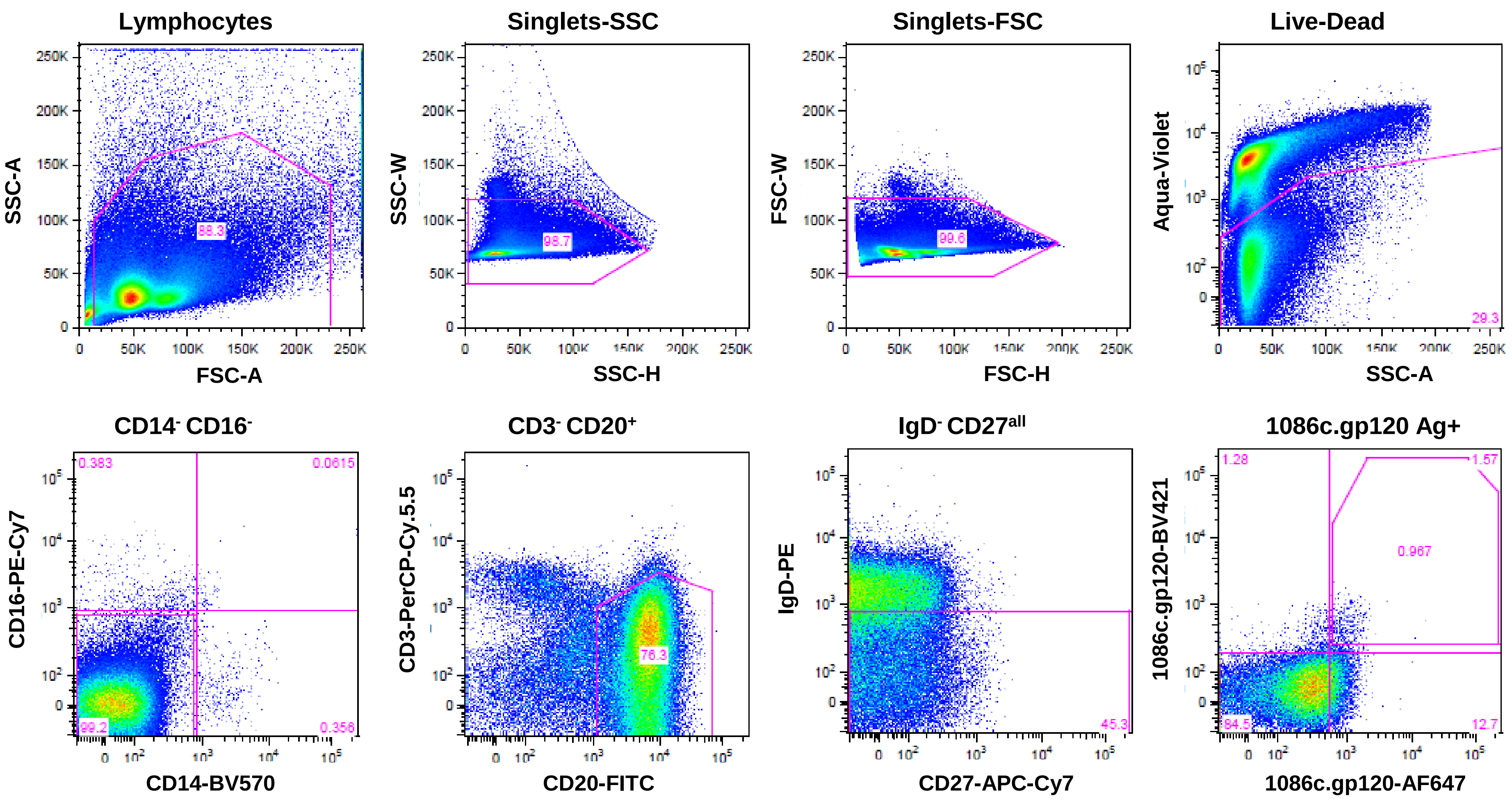

S1 Fig

G. Extended Interval group, animal 45435, Spleen (necropsy), representative data of 3 independent sort, gating strategy: lymphocytes → Singlets (SSC or FSC) → Live-dead → CD14-CD16- → CD3-CD20+ → IgD-CD27all → 1086c.gp120 double positive

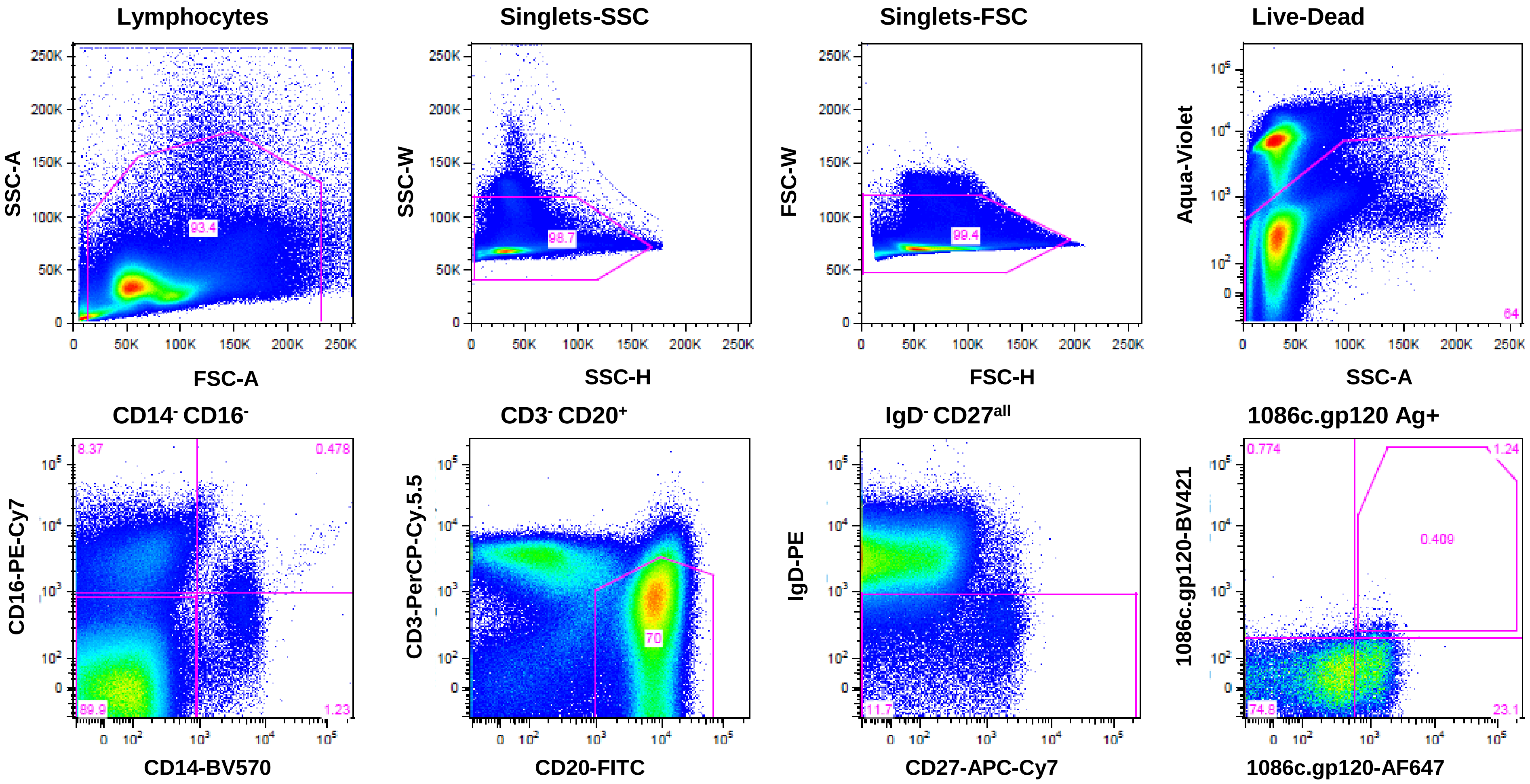

S1 Fig

H. Extended Interval group, animal 45441, Oral LN (submandibular, necropsy), representative data of 4 independent sort, gating strategy: lymphocytes → Singlets (SSC or FSC) → Live-dead → CD14-CD16- → CD3-CD20+ → IgD-CD27all → 1086c.gp120 double positive

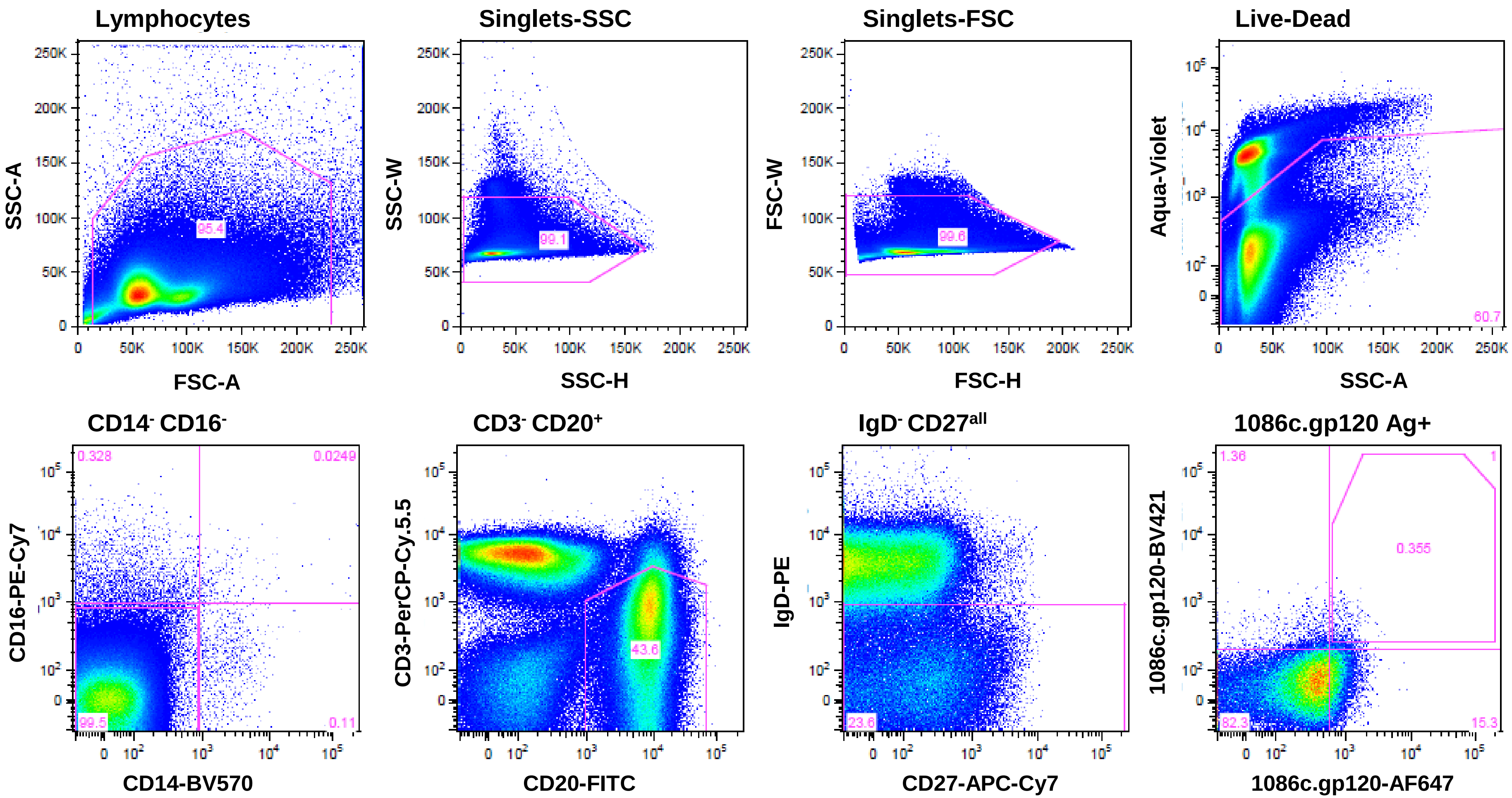

S1 Fig

I. Extended Interval group, animal 45448, Colon (necropsy), representative data of 3 independent sort, gating strategy: lymphocytes → Singlets (SSC or FSC) → Live-dead → CD14-CD16- → CD3-CD20+ → IgD-CD27all → 1086c.gp120 double positive

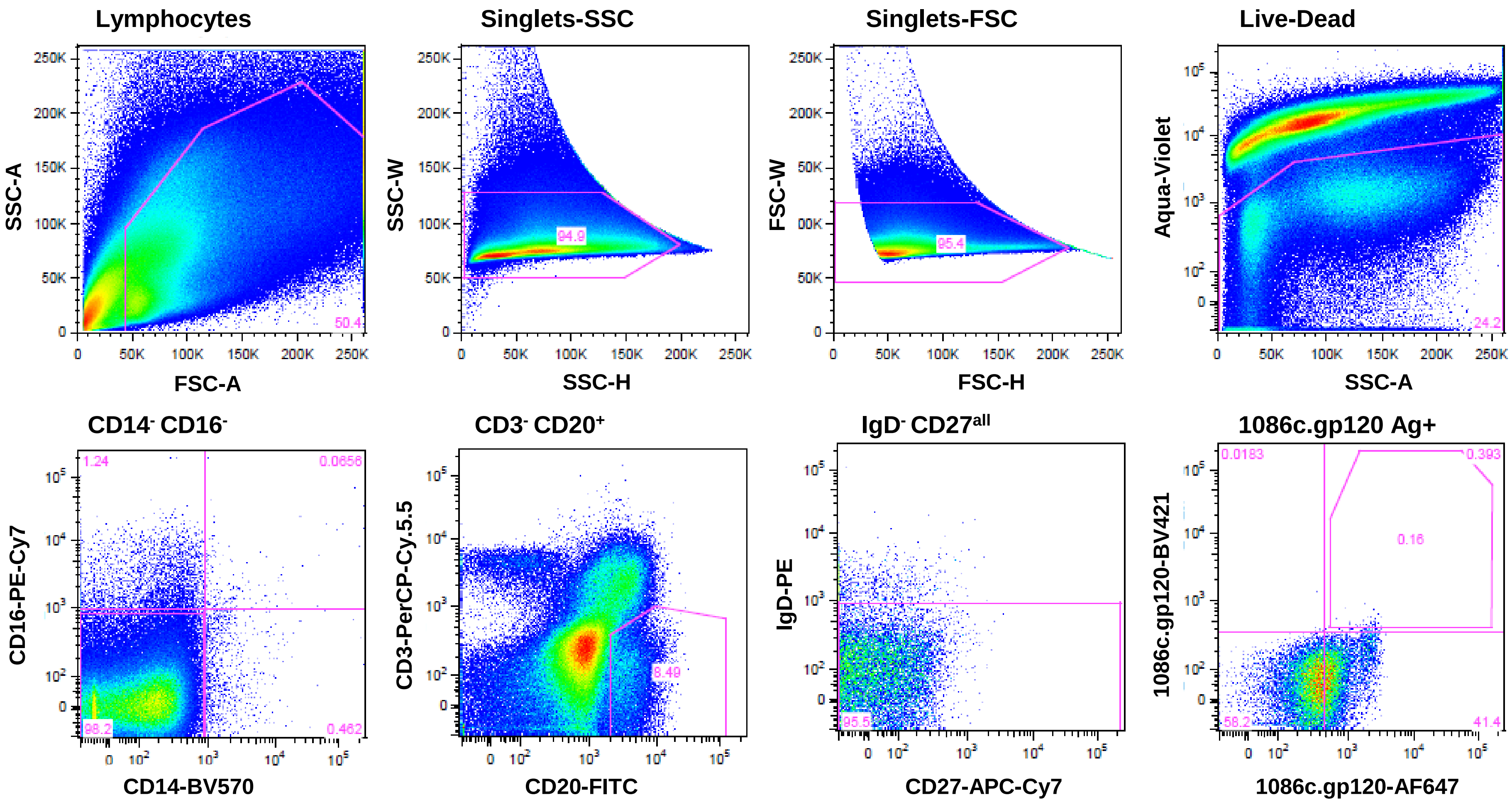

S1 Fig

J. 3M-052-SE group, animal 45838, PBMC (W12), gating strategy: lymphocytes → Singlets (SSC or FSC) → Live-dead → CD14-CD16- → CD3-CD20+ → IgD-CD27all → 1086c.gp120 double positive

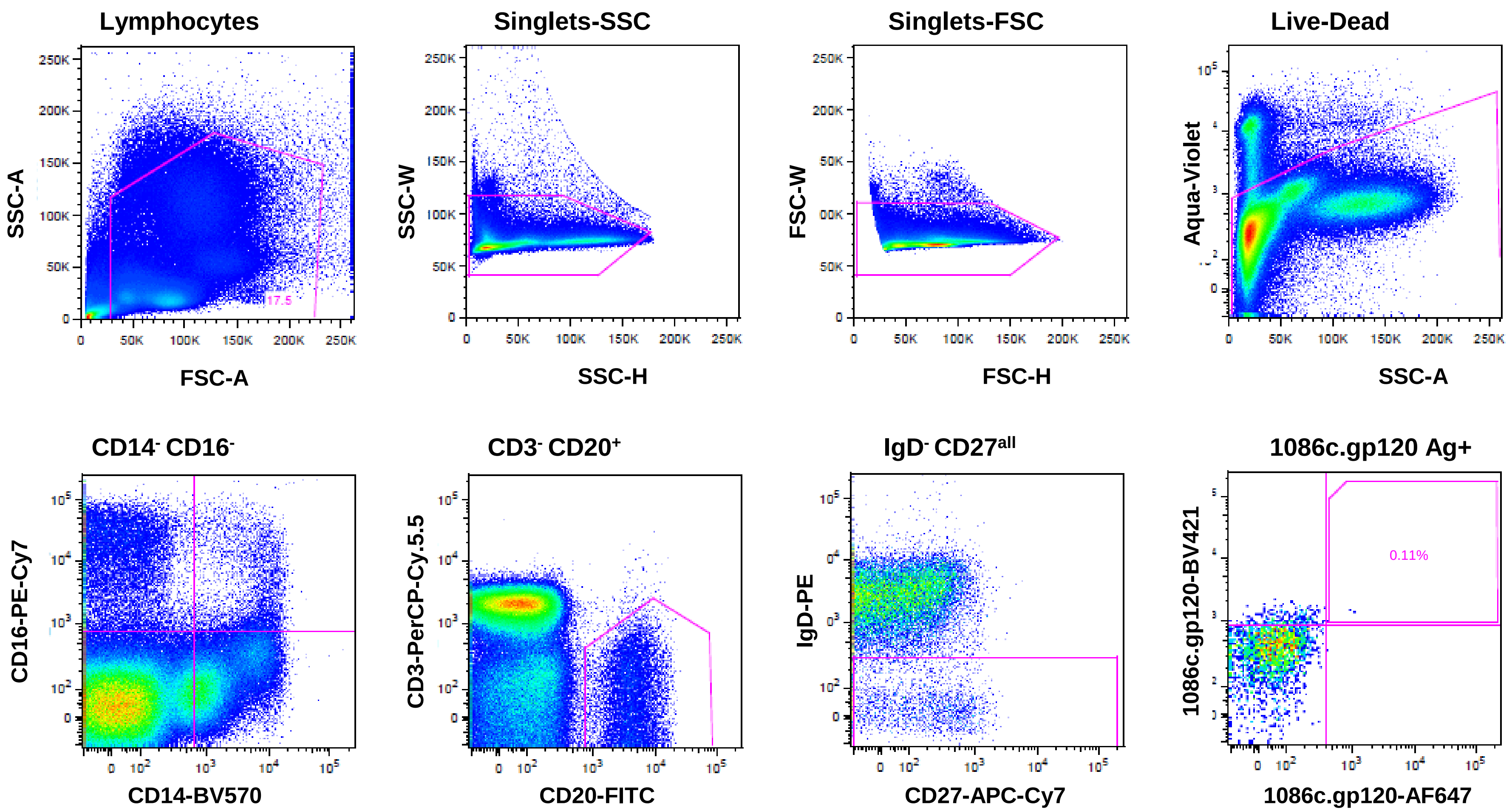

S1 Fig 1A-J. Representative flow cytometry analyses for single cell HIV Env-specific memory B cell sorting from PBMC and tissues of infants by vaccination groups. Percentage of memory B cells (CD20+CD27+IgD-), which were reactive to HIV 1086c gp120 K160N tagged with two colors-BV421 and AF647 (double positive cells) indicated in the upper right quadrant. Gates were drawn based on isotype controls and fluorescent minus one (FMO) controls.

S2 Fig

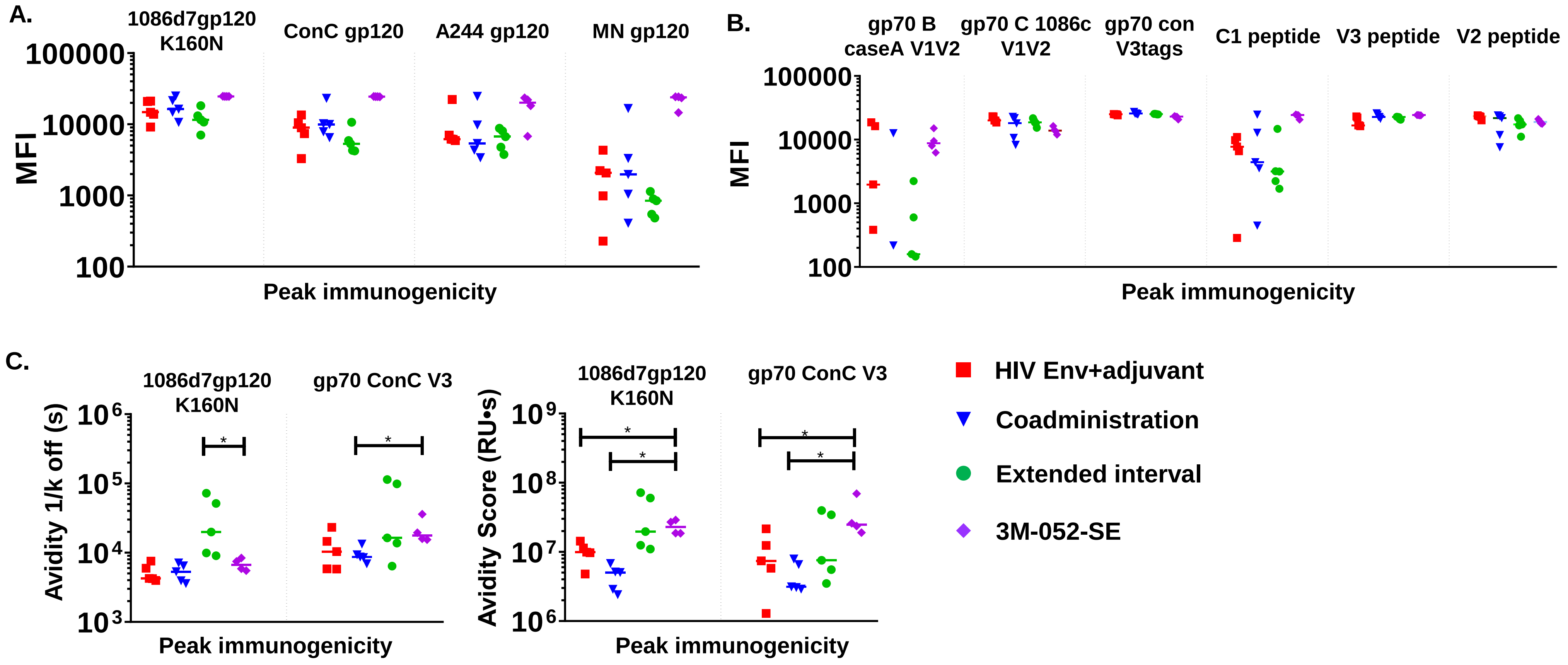

S2 Fig. Characterization of vaccination-induced plasma envelope IgG responses in infant rhesus macaques. (A) Cross clade HIV gp120-specific IgG responses at peak immunogenicity by luminex assay (BAMA). (B) Variable and conserved epitopes-specific IgG responses at peak immunogenicity by BAMA. (C) The 3M-052 group antibody responses had higher avidity strength against most of the tested antigens. Avidity 1/k off (the inverse of the dissociation rate) was plotted as a measure of the strength of binding and avidity scores which take into consideration the magnitude are also shown. Statistical analyses were performed with GraphPad Prism, * denoted significant p-values of <0.05 by a non-parametric Mann-Whitney test.

S1 Table. Immunogenetic characteristics of isolated envelope (Env)-reactive mAbs of Env-vaccinated infant monkeys based on human immunoglobulin database analysis. A total of 39 pairs of potentially Env-reactive mAbs were isolated from the four vaccination groups across several anatomic compartments. Frequency of gene usage, percent somatic hypermutation, and complementarity-region 3 (CDR3) length are displayed for the heavy and light chains for each mAb along with the isotype and epitope specificity.

Animal ID	Group	Tissue	IgH ID	V_H gene	D_H gene	J_H gene	HC % SHM	HC CDR3 length	Ig Isotype	IgL ID	V_L/V_K gene	J_L/J_K gene	Specificity
45521	HIV Env+adjuvant	Spleen	H020465	4~4*07	2~OF15*2/inv	3*01	5.31	23	IgG	K020382	1~33*01	2*03	Undetermined
45521	HIV Env+adjuvant	Retropharyngeal LN	H914640	4~4*07	3~3*01	3*01	6.14	23	IgA	K907482	1~33*01	2*03	Undetermined
45521	HIV Env+adjuvant	Retropharyngeal LN	H914648	4~4*07	3~3*01	3*01	6.65	23	IgG	K907485	1~33*01	2*03	Undetermined
45521	HIV Env+adjuvant	Retropharyngeal LN	H914649	4~4*07	5~12*01	3*01	5.88	23	IgG	K907486	1~33*01	2*03	Undetermined
45522	HIV Env+adjuvant	Spleen	H020414	4~61*03	6~13*01	4*02	10.03	13	IgG	L020264	1~51*02	3*02	Undetermined
45083	Coadministration	Spleen	H020400	4~59*01	6~19*01	4*02	6.17	13	IgM	L020253	1~40*01,02	2*01	V1V2
45083	Coadministration	Mediastinal LN	H020405	4~59*01,02	3~16*01,02	4*02	4.52	17	IgG	K020331	1D~16*01	2*03,04	V1V2
45083	Coadministration	Mediastinal LN	H020405	4~59*01,02	3~16*01,02	4*02	4.52	17	IgG	L020258	2~23*02	1*01	V3
45083	Coadministration	Mediastinal LN	H020407	4~b*02	5~12*01	4*02	8.41	13	IgG	L020260	2~8*01	1*01	V3
45083	Coadministration	Mediastinal LN	H020408	4~39*06	1~26*01	4*02	6.38	13	IgG	L020260	2~8*01	1*01	Undetermined
45091	Coadministration	Axillary LN	H020381	3~73*01,02	2~2*02/inv	6*02	10.15	14	IgG	L020241	11~55*01	2*01	Undetermined
45091	Coadministration	Axillary LN	H020387	4~59*01	4~4*01	4*02	4.74	17	IgG	K020324	1~39*01	4*01	V3
45435	Extended Interval	Mediastinal LN	H020422	4~59*01	3~3*01,02	3*01,02	6.19	18	IgA	K020341	1~13*02	4*01	V1V2
45435	Extended Interval	Mediastinal LN	H020425	4~59*01,02	3~OR15*3	5*01,02	7.63	16	IgG	K020344	1~33*01	2*03	CD4 binding site
45435	Extended Interval	Mediastinal LN	H020426	3~72*01	1~OR15*1	6*02	8.30	14	IgM	L020282	11~55*01	2*01	V3
45435	Extended Interval	Mediastinal LN	H020430	3~11*01	1~7*01/inv	5*01	10.61	17	IgG	K020348	1/OR2~0*01	2*03	V1V2
45435	Extended Interval	Mediastinal LN	H020431	3~64*02	4~17*01	5*01	9.40	12	IgG	K020349	3~11*01	1*01	V1V2
45435	Extended Interval	Mediastinal LN	H020445	4~4*02	4~4*01	4*02	6.53	16	IgG	K020359	1D~16*01	2*03	V3
45435	Extended Interval	Mediastinal LN	H020420	4~61*05	6~13*01	4*02	11.08	16	IgG	L020280	5~39*01	2*01	V3
45435	Extended Interval	Spleen	H020461	3~73*01,02	2~15*01	4*02	9.65	17	IgG	K020380	1~12*01,02	2*03	Undetermined
45441	Extended Interval	Spleen	H020449	3~21*01,02	1~IR1*01	1*01	6.05	29	IgG	K020361	1D~16*01	1*01	Undetermined
45441	Extended Interval	Spleen	H020452	4~59*01	3~3*01	4*02	7.18	18	IgG	L020292	1~51*02	7*01	CD4 binding site
45441	Extended Interval	Spleen	H020450	3~21*01,02	3~22*01	1*01	7.14	15	IgG	L020290	3~21*01	6*01	Undetermined
45441	Extended Interval	Submental LN	H914598	4~59*01	3~3*01	6*02	7.41	18	IgA	K907464	2~28*01	1*01	Undetermined
45838	3M-052-SE	PBMC	H020493	3~43*02	6~13*01	4*02	5.31	10	IgG	L020321	3~19*01	2*01	Undetermined
45840	3M-052-SE	PBMC	H020481	4~61*01,08	3~3*01,02	4*02	6.32	13	IgG	K020401	2~30*01	1*01	V3
45840	3M-052-SE	PBMC	H020485	4~39*06	3~3*01,02	4*02	6.12	13	IgG	K020402	2~40*01	1*01	V3
45840	3M-052-SE	PBMC	H020488	3~43*02	6~13*01	4*02	4.77	10	IgG	K020402	2~40*01	1*01	Undetermined
45851	3M-052-SE	PBMC	H020495	3~15*08	0~IR*01C	3*01	7.03	11	IgG	K020407	2~40*01	1*01	Undetermined
45851	3M-052-SE	PBMC	H020494	4~39*06	3~3*01,02	4*02	6.89	13	IgG	K020409	2~30*01	1*01	Undetermined
45851	3M-052-SE	PBMC	H020500	4~59*01	6~13*01	1*01	4.21	10	IgA	L020324	5~48*01	1*01	V3
45851	3M-052-SE	PBMC	H020498	5~51*01	3~10*01	5*01	4.15	13	IgG	L020327	1~50*01	2*01	V3
45851	3M-052-SE	PBMC	H020499	3~43*02	2~8*02	4*02	5.61	16	IgG	L020326	6~57*01	7*01	Undetermined
45851	3M-052-SE	PBMC	H020505	4~59*01	1~1*01	1*01	4.21	10	IgM	L020329	6~57*01	7*01	V3
45851	3M-052-SE	PBMC	H020501	4~39*06	3~3*01,02	4*02	6.63	13	IgG	L020328	5~48*01	1*01	V3
45851	3M-052-SE	PBMC	H020504	3~43*02	6~13*01	4*02	5.31	10	IgG	L020329	6~57*01	7*01	Undetermined
45851	3M-052-SE	PBMC	H020506	4~39*06	3~3*01,02	1*01	6.38	13	IgE	L020333	6~57*01	7*01	V3
45851	3M-052-SE	PBMC	H020496	3~43*02	2~8*02	4*02	5.57	16	IgG	K020407	2~40*01	1*01	Undetermined
45851	3M-052-SE	PBMC	H020497	4~59*01	1~1*01	1*01	3.95	10	IgG	K020409	2~30*01	1*01	V3

S3 Fig

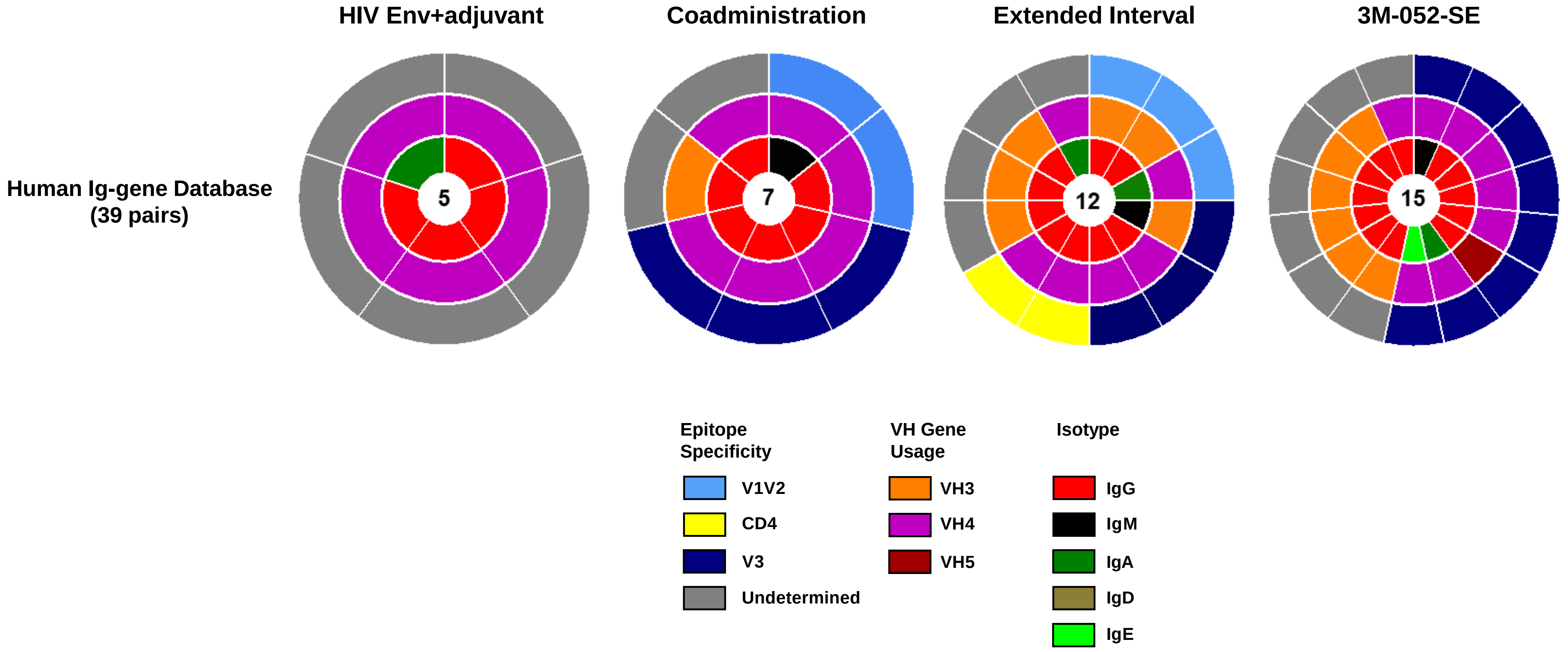

S3 Fig. Analyses of epitope specificity and immunogenetic characteristics of the Env-specific functional heavy- and light-chains of 39 vaccine-elicited mAbs in infants using human Ig-gene database. Initial analysis with human immunoglobulin (Ig) database indicated a total of 39 heavy- and light-chain pairs isolated from antigen-specific memory B cells across different vaccine groups. Epitope specificity, VH gene family usage, and isotype distribution of identified functional heavy- and light-chain pairs were similar across vaccine groups. Epitope specificity, VH gene family usage, and isotype distribution of identified functional heavy and light chains are displayed in concentric circles. The number of mAbs per group is displayed in the center.

S4 Fig

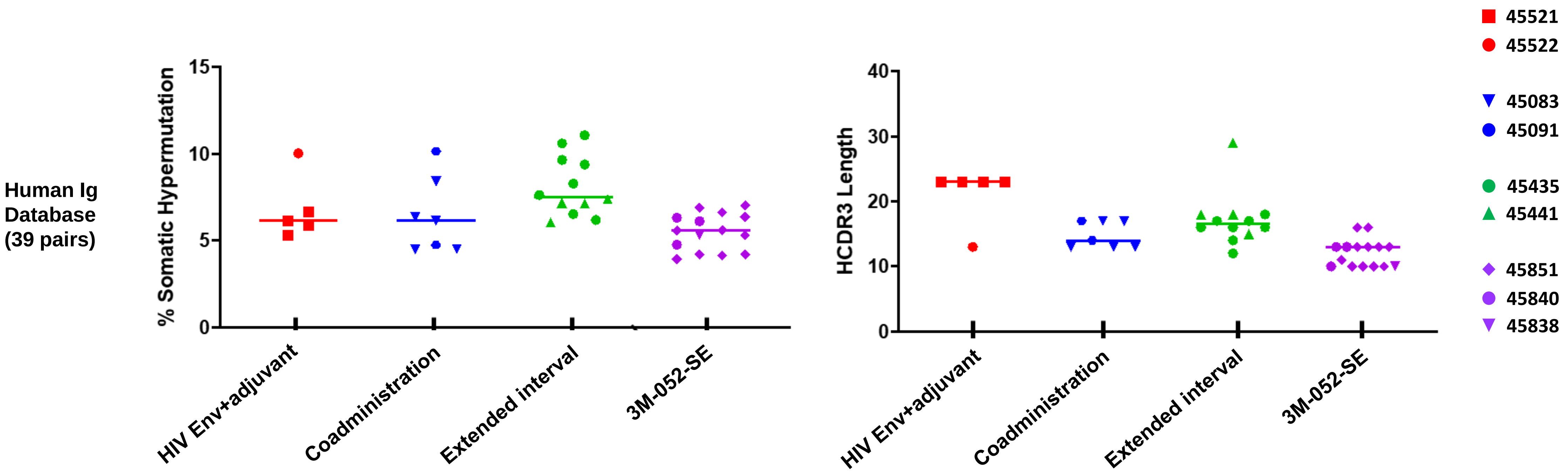

S4 Fig. Frequency of somatic hypermutation and heavy chain complementarity-determining region 3 (HCDR3) length of vaccine-elicited Env-reactive functional heavy- and light-chains identified using rhesus Ig sequence database. Analysis of percent somatic hypermutation frequency and HCDR3 lengths for Env-reactive heavy and light chains pairs (39 mAb pairs) from infant antigen-specific B cells based on human immunoglobulin (Ig) sequence database. Horizontal lines indicated median values of individual groups. Corresponding functional heavy and light chains isolated from individual infants are denoted by symbols.
